# *Xanthomonas campestris* utilizes IAA to regulate its viability and virulence by altering the production of BCAAs and ROS

**DOI:** 10.1002/mlf2.70033

**Published:** 2025-10-27

**Authors:** Sinan Li, Kai Song, Ying Cui, Lin Li, Minglei Zhang, Ya‐Wen He

**Affiliations:** ^1^ State Key Laboratory of Microbial Metabolism, Joint International Research Laboratory of Metabolic and Developmental Sciences, School of Life Sciences and Biotechnology Shanghai Jiao Tong University Shanghai China

**Keywords:** BCAA, indole‐3‐acetic acid, reactive oxygen species, virulence, *Xanthomonas campestris* pv. *campestris*

## Abstract

Indole‐3‐acetic acid (IAA) is an important plant hormone that regulates a variety of physiological processes in plants, and it is also produced by some microbes. However, the biosynthesis and roles of IAA in microorganisms, particularly in plant pathogens, remain to be determined. In this study, the plant pathogen *Xanthomonas campestris* pv. *campestris* (Xcc) strain XC1 was shown to produce IAA via an l‐tryptophan ( l‐Trp)‐dependent pathway. The intermediate metabolite indole‐3‐ethanol and Xcc1569 encoding aromatic amino acid aminotransferase were shown to be partially involved in the uncharacterized sub‐pathway in an l‐Trp‐dependent IAA biosynthetic pathway. IAA positively regulated the viability of XC1, as indicated by its colony‐forming units (CFUs), extracellular polysaccharide production, protease activity, and virulence on cabbage. IAA also negatively regulated reactive oxygen species (ROS) production in XC1. Furthermore, RNA‐Seq revealed a gene cluster, *ilvCGM*‐*leuA*, encoding the products responsible for branched‐chain amino acid (BCAA) biosynthesis, which was negatively regulated by IAA. High‐performance liquid chromatography (HPLC) analysis showed that IAA negatively regulated valine and leucine production. Deletion of *ilvC* significantly increased the CFUs and reduced the ROS levels of XC1. Exogenous BCAA addition to mutant strain Δ*ilvC* restored the CFU and ROS levels to those of wild‐type strain XC1. These results revealed an IAA signaling cascade in XC1 that involved *ilvCGM*‐*leuA*, BCAA production, ROS production, and colony formation. These IAA‐regulated phenotypes contributed to the virulence of Xcc in host plants. Overall, these results explain IAA‐mediated plant–Xcc interactions and underscore the potentially significant role of IAA in microbial physiology.

## Impact statement

Indole‐3‐acetic acid (IAA) is a critical phytohormone. Accumulating evidence suggests that IAA may act as a messenger for microbe–plant communication. In this study, the plant pathogen *Xanthomonas campestris* pv. *campestris* (Xcc) was shown to contain a novel l‐tryptophan‐dependent IAA biosynthetic pathway. Xcc utilizes the self‐produced IAA or the host plant‐derived IAA to promote its virulence toward cabbage. The IAA signaling cascade in XC1 involved the gene cluster *ilvCGM*‐*leuA*, branched‐chain amino acid production, reactive oxygen species production, and cell viability. These findings reveal IAA‐mediated plant–Xcc interactions and underscore the potentially significant role of IAA in microbial physiology.

## INTRODUCTION

Indole‐3‐acetic acid (IAA), the most abundant natural auxin in plants, controls a broad range of physiological processes in plant growth and development, including cell enlargement and division, tissue differentiation, and responses to environmental stimuli^1^, ^2^, ^3^. IAA not only acts as a coordinating hormone between plant cells but also as a trigger of environmentally controlled plant development and as a messenger for microbe–plant communication^4^, ^5^. Understanding IAA‐mediated plant–microbe interactions is still a subject of intense investigation among the scientific community.

IAA production is not only limited to plant cells but also observed in microbes, such as bacteria, yeast, and filamentous fungi^6^, ^7^. A wide range of rhizosphere bacteria, including *Acetobacter*, *Acinetobacter*, *Arthrobacter*, *Azotobacter*, *Azospirillum*, *Bacillus*, *Bradyrhizobium*, *Burkholderia*, *Herbaspirillum*, *Klebsiella*, *Mesorhizobium*, *Paenibacillus*, *Pantoea*, *Pseudomonas*, *Rhizobium*, *Rhodococcus*, *Rouxiella*, *Serratia*, *Stenotrophomonas*, and *Streptomyces* species, have been reported to produce IAA^7^, ^8^, ^9^, ^10^, ^11^, ^12^, ^13^. The ability to produce IAA is recognized as a molecular marker for plant growth‐promoting microbe identification^14^, ^15^. Microbial IAA synthesis is generally categorized into two main pathways: l‐tryptophan (l‐Trp)‐dependent and l‐Trp‐independent. l‐Trp serves as the primary precursor for IAA biosynthesis in l‐Trp‐dependent pathway, which can be broadly grouped into indole‐3‐acetamide (IAM), indole‐3‐pyruvic acid (IPA), indole‐3‐acetonitrile (IAN), tryptamine (TAM), and l‐Trp side‐chain oxidase (TSO) sub‐pathways based on the downstream intermediate metabolites^16^, ^17^. A few microbes, such as the nitrogen‐fixing bacterium *Azospirillum brasilense* and endophytic fungus *Cyanodermella asteris*, have also been recognized as producing IAA via l‐Trp‐independent pathway, mainly utilizing indole‐3‐glycerol phosphate or indole as fundamental precursors^17^, ^18^.

Although IAA biosynthesis has been well studied in rhizosphere bacteria, much less attention has been paid to plant pathogens. *Agrobacterium tumefaciens* induces crown gall tumors by transferring ∼20 kb of single‐stranded transfer DNA (T‐DNA) into plant cells^19^. The T‐DNA encodes enzymes that convert l‐Trp into IAA via the indole acetamide (IAM) pathway^20^. The bacterial pathogen *Pseudomonas syringae* strain DC3000 has been shown to synthesize IAA via an indole‐3‐acetaldehyde dehydrogenase pathway^21^. The Gram‐positive phytopathogen *Rhodococcus fascians* has been shown to produce IAA mainly through the IPA pathway to incite leafy galls on a wide range of plants^22^. Bacterial pathogens of rice *Xanthomonas oryzae* pv. *oryzae* (Xoo) and *X*. *oryzae* pv. *oryzicola* (Xoc) have been shown to secrete IAA, which, in turn, may cause rice to synthesize its own IAA at the infection site^23^. Recently, Xoc has been shown to produce IAA via a nitrilase‐dependent biosynthesis pathway^24^. Nevertheless, the biosynthesis and roles of IAA in phytopathogen growth, development, and virulence remain to be fully understood.

Cruciferous vegetables have amassed widespread popularity and are now cultivated around the world. They are high in vitamins A, C, and K and dietary fiber. These vegetables are also unique in the sulfur‐containing compounds called glucosinolates, which have been linked to a long list of health benefits^25^, ^26^. Black rot is a potentially lethal bacterial disease that affects cruciferous vegetables. *Xanthomonas campestris* pv. *campestris* (Xcc), the causal agent of black rot^27^, ^28^, is a vascular pathogen that enters plants through leaf margin hydathodes, stomata, and wounds^29^, ^30^. Xcc infection has been shown to induce IAA biosynthesis in *Arabidopsis thaliana* Columbia leaf tissue^31^. It remains unknown if Xcc produces IAA and how Xcc induces IAA biosynthesis inside the host plants, and it is not clear if IAA affects Xcc physiology and virulence in host plants.

This study aimed to (1) identify the pathway through which Xcc produces IAA; (2) determine the effects of IAA production on the colonization and virulence of Xcc in host plants; and (3) determine the molecular mechanisms responsible for IAA‐dependent phenotypes. Through a combination of genetic and chemical analyses, we showed that Xcc used an l‐Trp‐dependent IAA biosynthetic pathway and that indole‐3‐ethanol (TOL) was one of the key intermediates in this pathway. IAA positively regulated Xcc viability, virulence factor production, and virulence in host plants and negatively regulated reactive oxygen species (ROS) levels and the biosynthesis of branched‐chain amino acids (BCAAs). The IAA‐regulated cell viability was probably mediated by the BCAA signaling pathway. This study reveals the mechanisms involved in IAA‐mediated plant–Xcc interactions and underscores the potentially significant role of IAA in microbial physiology.

## RESULTS

### IAA biosynthesis in Xcc strain XC1 is subject to culture conditions and growth stage

**Figure 1:**
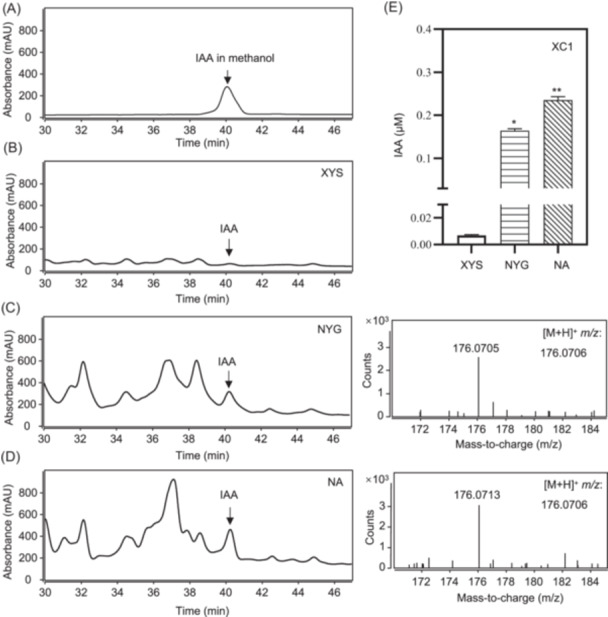
*Xanthomonas campestris* strain XC1 produces detectable indole‐3‐acetic acid (IAA) under laboratory growth conditions. (A) High‐performance liquid chromatography (HPLC) analysis of IAA dissolved in methanol (50 μM). (B) HPLC analysis of IAA extracts from XYS culture at 12 hours post inoculation (hpi). (C, D) HPLC and ultrahigh‐performance liquid chromatography‐time‐of‐flight mass spectrometry (UPLC‐TOF‐MS) analysis of IAA extracts from NYG and NA cultures at 12 hpi. (E) Quantitative analysis of the relative IAA level in XYS, NYG, and NA cultures at 12 hpi. Three independent experiments were conducted, and data are expressed as the mean ± SD. *p < 0.05; **p < 0.01.

In this study, three media, Xylem‐Sucrose (XYS), Nutrient Yesst Glycerol (NYG), and Nutrient Broth‐A (NA), were used to culture XC1. XYS medium is a specially designed medium that mimics the within‐plant growth conditions during Xcc infection. Both NYG and NA are rich media used for Xcc culturing^28^. To quantitatively evaluate IAA biosynthesis in XC1 culture, a high‐performance liquid chromatography (HPLC)‐based method was established in this study (Figure S1). IAA was extracted from XC1 cultures grown in XYS, NYG, and NA media at 12 hours post inoculation (hpi), respectively. The extracts were submitted for HPLC, and a peak observed at 40.1 min corresponded to IAA (Figure 1A–D). The IAA identity in the peak collection was further verified by ultrahigh‐performance liquid chromatography‐time‐of‐flight mass spectrometry (UPLC‐TOF‐MS) analysis (Figure 1C,D). Comparatively, the IAA level of XC1 in the XYS medium at 12 hpi was significantly lower than that in NYG and NA medium (Figure 1E).

**Figure 2:**
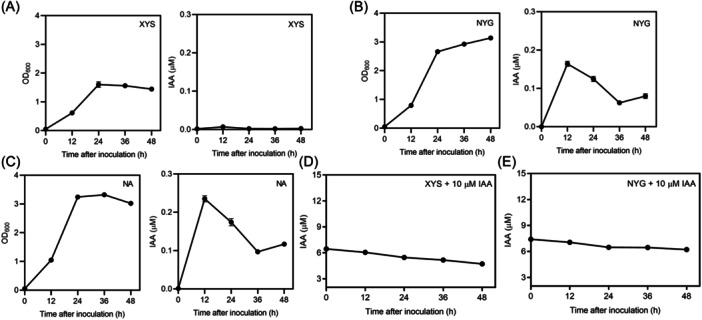
IAA levels of the XC1 strain in XYS, NYG, and NA media over time. (A–C) Growth and IAA level of XC1 in XYS (A), NYG (B), and NA (C) medium. (D, E) IAA Level of the XC1 strain in XYS (D) and NYG (E) medium supplemented with 10 μM IAA. Three independent experiments were conducted, and data are expressed as the mean ± SD of three independent assays.

Furthermore, IAA production was determined over time in XC1 during growth in XYS, NYG, and NA media for 12−48 h. The IAA level reached the highest level at 12 hpi and then declined dramatically in the XC1 cultures of three media (Figure 2A–C). Since the IAA level decreased slowly at 12–48 hpi when IAA was exogenously added to the XYS and NYG medium at a final concentration of 10 μM (Figure 2D,E), the dramatic decline in the IAA level in the XC1 culture may be due to the shortage of the IAA biosynthetic substrate and IAA metabolism at 12–36 hpi.

### XC1 produces IAA via an l‐Trp‐dependent pathway

**Figure 3:**
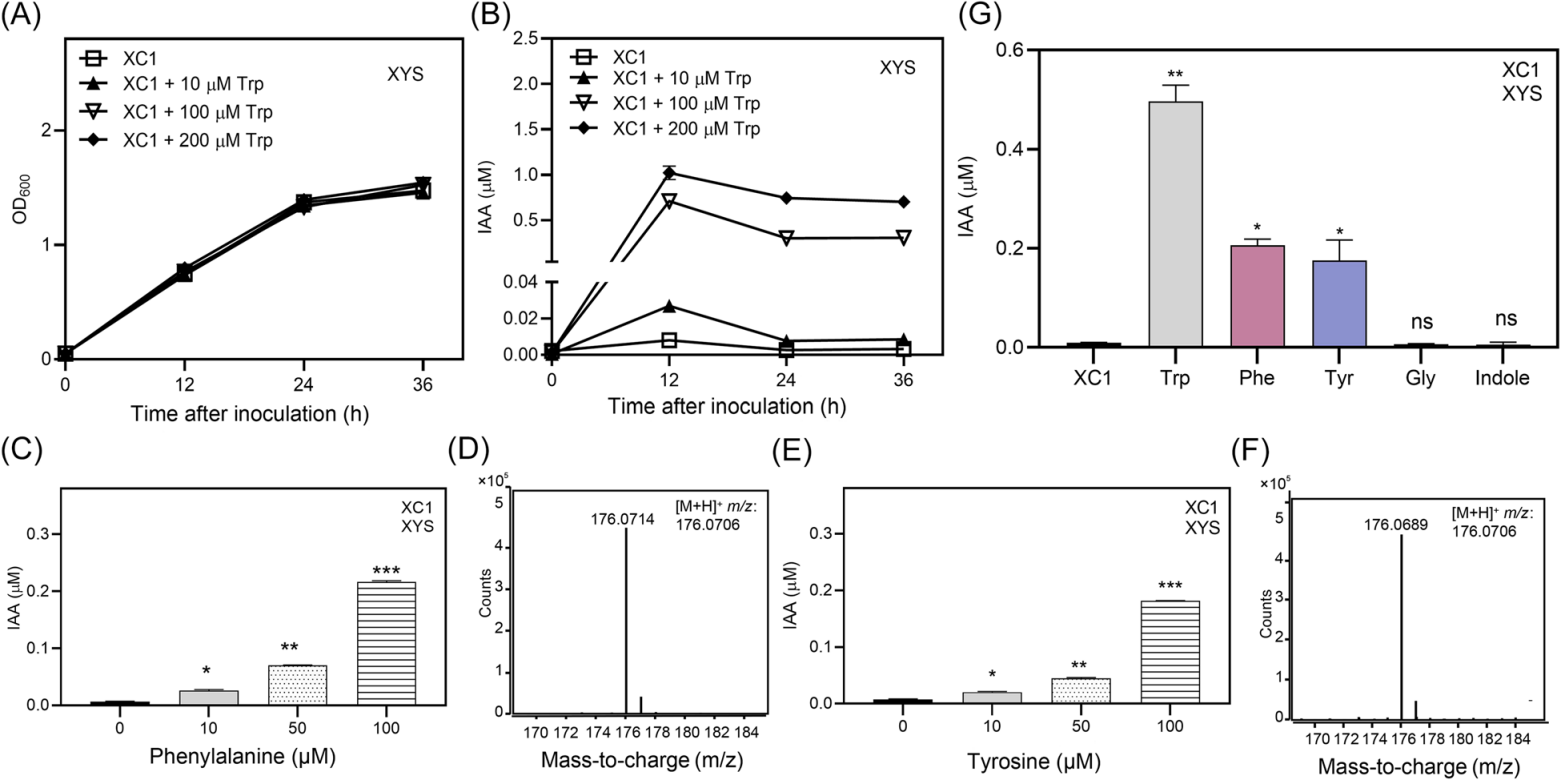
XC1 uses l‐tryptophan (l‐Trp), phenylalanine (Phe), and tyrosine (Tyr) to synthesize IAA. (A, B) Growth–time curve (A) and IAA levels (B) of the XC1 strain in XYS medium supplemented with 10–200 μM l‐Trp. (C, E) IAA levels of strain XC1 in XYS medium supplemented with 10–100 μM Phe (C) and l‐Tyr (E). (D, F) UPLC‐TOF‐MS analysis of Phe‐ (D) and Tyr‐ (F) dependent IAA. (G) Effect of 100 μM l‐Trp, Phe, Tyr, Glycine (Gly), and indole on IAA biosynthesis. Three independent experiments were conducted, and data are expressed as the mean ± SD. *p < 0.05; **p < 0.01; ***p < 0.001; ns, not significant.

l‐Trp has been well defined as a precursor for IAA biosynthesis in plants and a wide range of microorganisms^16^, ^32^. In this study, to investigate the IAA biosynthetic pathway in XC1, XYS medium was supplemented with 10–200 μM l‐Trp. When grown in l‐Trp‐containing XYS media, IAA production was significantly increased in a l‐Trp concentration‐dependent manner, reaching 0.03, 0.71, and 1.02 μM in the presence of 10, 100, and 200 μM l‐Trp, respectively, at 12 hpi (Figure 3A,B). In contrast, the addition of l‐Trp to the XYS medium without XC1 failed to produce IAA (Figure S2). These results suggest that the increase in the IAA level in the XC1 culture was derived from l‐Trp.

In addition to l‐Trp, both phenylalanine (Phe) and tyrosine (Tyr) are also aromatic amino acids. In this study, we found that the addition of aromatic amino acids Phe and Tyr (10–100 μM) to XYS culture of XC1 also significantly increased IAA biosynthesis in a concentration‐dependent manner (Figure 3C,E). The Phe‐ or Tyr‐dependent IAA production was further verified by TOF‐MS analysis (Figure 3D,F). However, the IAA‐promoting effects of Phe and Tyr were significantly lower than those of l‐Trp (Figure 3G). In contrast, the amino acid glycine (Gly) had no promoting effect on IAA biosynthesis in the XYS medium (Figure 3G).

Indole and indole‐3‐glycerol phosphate have been shown to be fundamental precursors of the l‐Trp‐independent IAA biosynthetic pathway in several microorganisms^17^, ^18^. In this study, 100 μM indole was added to XYS media for XC1 culture, but no significant increase in the IAA level was observed (Figure 3G).

### XC1 contains unique l‐Trp‐dependent IAA biosynthetic pathways

**Figure 4:**
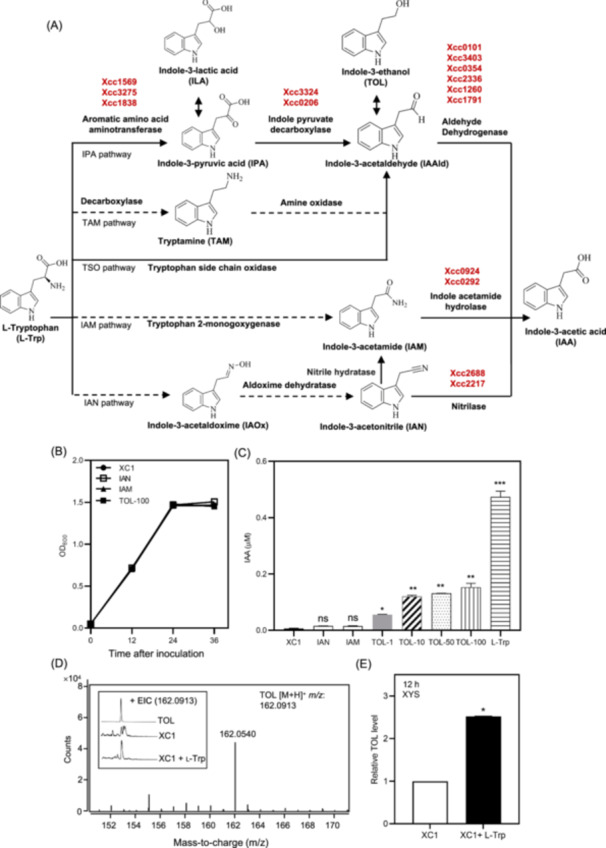
Unique l‐Trp‐dependent IAA biosynthesis in XC1**.** (A) Proposed l‐Trp‐dependent IAA biosynthetic sub‐pathways in bacteria. (B) Growth curve of XC1 in XYS medium supplemented with 100 μM IAN, IAM, or TOL. (C) IAA levels in XC1 cultured in XYS medium supplemented with 100 μM l‐Trp, IAN, IAM, or 1–100 μM TOL at 12 hpi. (D) UPLC‐TOF‐MS analysis of l‐Trp‐dependent TOL. (E) Relative TOL level of the XC1 culture in the absence and presence of 100 μM l‐Trp. Three independent experiments were conducted, and data are expressed as the mean ± SD. *p < 0.05; **p < 0.01; ***p < 0.001; ns, not significant. IAM, indole‐3‐acetamide pathway; IAN, indole‐3‐acetonitrile; IPA, indole‐3‐pyruvic acid pathway; TAM, tryptamine pathway; TOL, indole‐3‐ethanol; TSO, tryptophan side‐chain oxidase pathway.

Five putative sub‐pathways in the l‐Trp‐dependent IAA biosynthesis pathway have been proposed in various bacterial strains (Figure 4A). In this study, to verify the sub‐pathways in XC1 IAA biosynthesis, the bacterial and fungal genes encoding the putative enzymes involved in the proposed five sub‐pathways were identified (Figure 4A). They were used as templates for BlastP analysis and domain organization analysis in Xcc strain ATCC33913. The analysis identified a total of 15 putative IAA biosynthetic genes, including 11 genes encoding aromatic amino acid aminotransferase, indole pyruvate decarboxylase and aldehyde dehydrogenase in the IPA pathway, 2 genes (Xcc0292 and Xcc0924) encoding indole acetamide hydrolase in the IAM pathway, and 2 genes (Xcc2688 and Xcc2217) encoding nitrilase in the IAN pathway (Figures 4A and S3). These genes were deleted in the strain XC1 and the resultant mutant strains were analyzed for growth and IAA production in XYS cultures supplemented with 100 μM l‐Trp. Sole deletion of these genes in XC1 had no significant effect on bacterial growth (Figure S4A). Deletion of Xcc1569 encoding aromatic amino acid aminotransferase in the IPA pathway significantly decreased 42.0% IAA production (Figure S4B); however, deletion of the putative genes encoding indole pyruvate decarboxylase and aldehyde dehydrogenase (*Xcc0101*, *Xcc0206*, *Xcc0354*, *Xcc1260*, *Xcc1791*, *Xcc3324*, and *Xcc3403*) in the same IPA pathway had no significant effect on IAA production (Figure S4B). Deletion of other genes encoding the key enzymes in IAM and IAN pathways (*Xcc0292*, *Xcc0924*, *Xcc2217*, and *Xcc2688*) also had no significant effect on IAA production (Figure S4B).

In this study, we also generated the overexpression mutants for all these 15 putative IAA biosynthetic genes via the plasmid pBBR‐1‐MCS in XC1. Overexpression of these genes except for Xcc0292 had no significant effect on bacterial growth (Figure S5A). Overexpression of Xcc1569 significantly increased IAA production by 48.2%, while overexpression of Xcc0292 in XC1 significantly decreased IAA production by 42.7% (Figure S5B). The decreased IAA production in the Xcc0292 overexpression strain occurred probably due to the decreased bacterial growth (Figure S5B). These results suggest that Xcc1569 is partially involved in the l‐Trp‐dependent IAA biosynthesis in XC1.

To further dissect the l‐Trp‐dependent IAA biosynthetic pathway in XC1, three previously identified intermediate metabolites, IAN, IAM, and TOL, were respectively added to XYS media for XC1 culture, and the IAA level was determined at 12 hpi. The addition of IAN, IAM, or TOL to XC1 culture had no significant effect on bacterial growth (Figure 4B). The addition of 100 μM IAN or 100 μM IAM had no significant effect on IAA production; however, the TOL addition significantly increased the IAA level in a concentration‐dependent manner (Figure 4C). The IAA level increased from 0.055 at 1.0 μM TOL to 0.153 at 100 μM TOL, representing a 2.78‐fold increase (Figure 4C). Comparatively, the level of TOL‐induced IAA biosynthesis is approximately one‐third of the l‐Trp‐induced one in XC1 (Figure 4C). Further, using the commercially available TOL as a control, UPLC‐Q‐TOF‐MS analysis detected TOL in the XYS medium of XC1 in the presence of 100 μM l‐Trp (Figure 4D). Quantitative analysis revealed that the TOL level in the presence of 100 μM l‐Trp increased 2.52‐fold compared to that in the absence of l‐Trp (Figure 4E). These results suggest that TOL is partially involved in l‐Trp‐dependent biosynthesis in XC1.

### IAA positively regulates EPS production, extracellular protease activity, and the virulence of XC1 on cabbage

**Figure 5:**
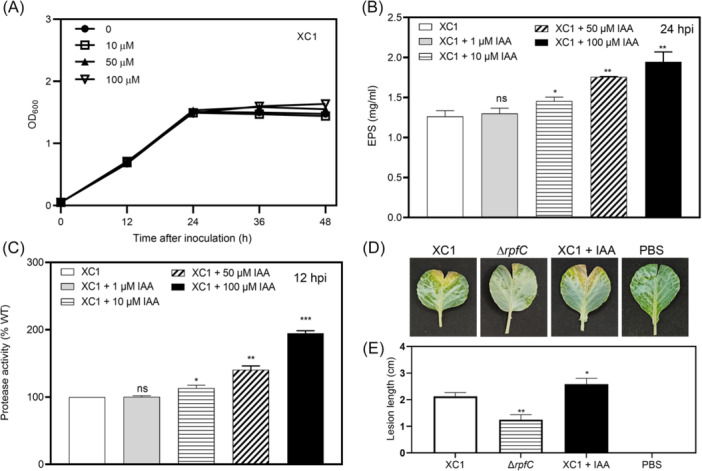
Effects of IAA on extracellular polysaccharide (EPS) production, extracellular protease activity, and virulence on cabbage. (A) XC1 growth in the presence of 10–100 μM IAA. (B) EPS production of Xcc strain XC1 in XYS medium or XYS medium supplemented with 1–100 μM IAA at 24 hpi. (C) Protease activity of XC1 in XYS medium or XYS medium supplemented with 1–100 μM IAA at 12 hpi. (D) Lesions on cabbage at 12 days post inoculation (dpi). XC1, Xcc wild type strain; Δ*rpfC*, rpfC deletion mutant; XC1 + IAA, XC1 strain treated by IAA; PBS, phosphate buffered saline. (E) Quantitative analysis of the lesion length caused by strains XC1, Δ*rpfC*, and IAA‐treated XC1 (XC1 + IAA) at 12 dpi on cabbage. Each treatment contains 12 cabbage leaves. Data are expressed as the mean ± SD. *p < 0.05; **p ≤ 0.01; ***p < 0.001; ns, not significant.

Xcc produces extracellular polysaccharides (EPSs) and a series of extracellular enzymes, including amylase, endoglucanase, polygalacturonate lyase, and protease, that collectively contribute to pathogenesis^33^, ^34^. In this study, EPS production and protease activity of XC1 in liquid XYS medium were determined in the absence and presence of 1–100 μM IAA. The addition of 10–100 μM IAA to XC1 culture had no significant effect on the bacterial growth, as indicated by the OD_600_ (Figure 5A). The EPS levels of XC1 at 24 hpi were 1.45 mg/ml at 10 μM IAA, 1.75 mg/ml at 50 μM IAA, and 1.9 mg/ml at 100 μM IAA (Figure 5B). These values were significantly higher than those found in the absence of IAA and at 1 μM IAA, with 1.26 and 1.30 mg/ml produced at 24 hpi, respectively (Figure 5B).

In the presence of 1, 10, 50, and 100 μM IAA, the extracellular protease activity of XC1 increased by 0.31%, 13.1%, 40.5%, and 94.7% at 12 hpi, respectively (Figure 5C). These results suggest that IAA positively regulates EPS production and protease activity in the XYS culture of XC1.

To investigate the role of IAA in the virulence of Xcc, XC1 was grown in an XYS liquid medium supplemented with 100 μM IAA for 12 h. The IAA‐treated XC1 (XC1 + IAA), and nontreated XC1 were used to inoculate cabbage. *rpfC* encodes a hybrid histidine kinase sensor for the quorum‐sensing signal diffusible signaling factor (DSF) in Xcc. The mutant strain Δ*rpfC* displayed significantly reduced virulence in cabbage^28^ and was used as a negative control in this study. At 12 days post inoculation (dpi), cabbage inoculated with the IAA‐treated XC1 strain showed a 21.7% increase in lesion length compared to that inoculated with the wild‐type strain of XC1 (Figure 5D,E). In contrast, the negative control strain Δ*rpfC* caused a significantly reduced lesion length than the wild‐type strain of XC1 (Figure 5D,E). These results suggest that IAA positively regulates the virulence of XC1 in cabbage.

### IAA prevents XC1 cell death at the late growth phases in XYS medium

**Figure 6:**
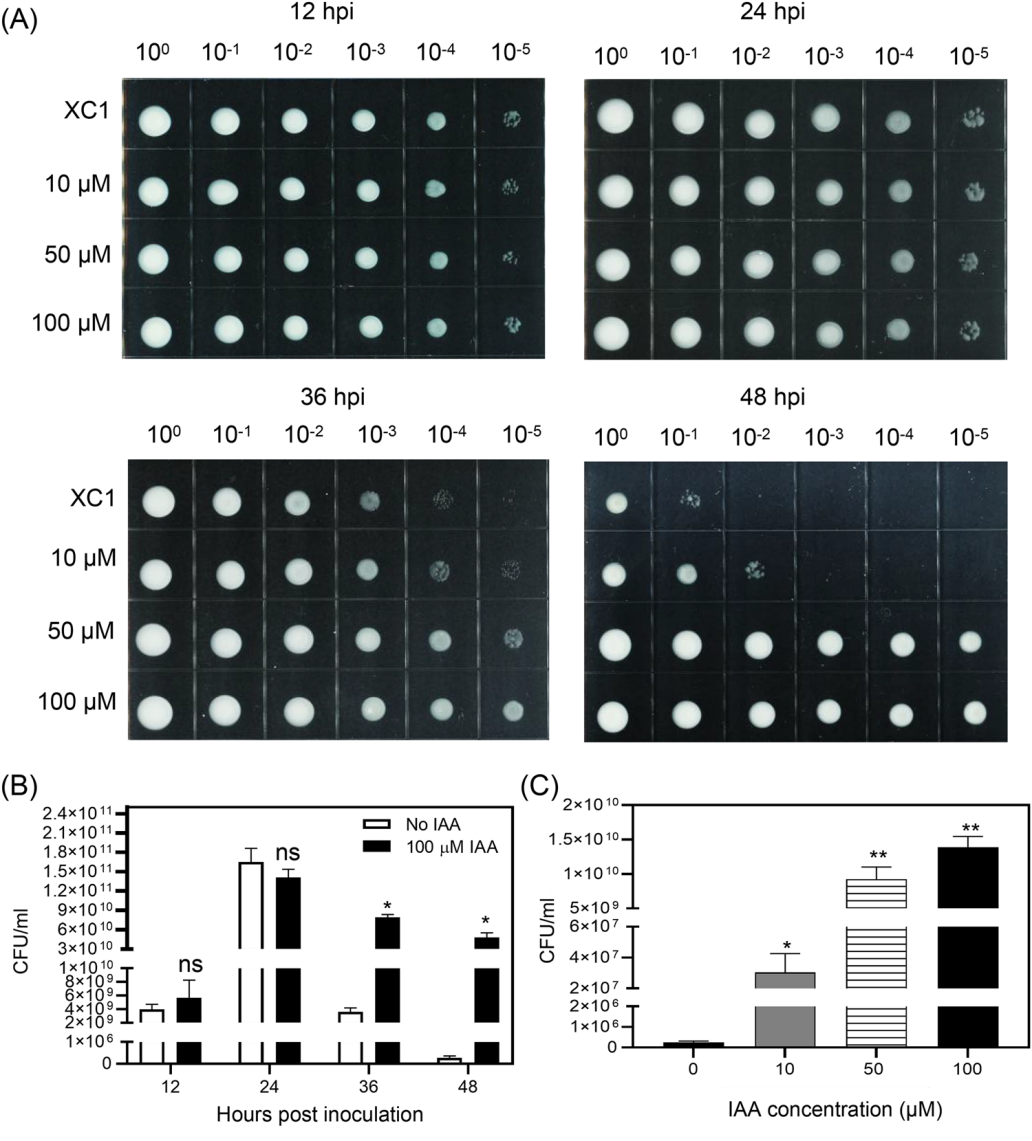
Effects of IAA on the number of colony‐forming units (CFUs) produced by XC1. (A) CFU assay for the XC1 strain grown in XYS medium supplemented with 0–100 μM IAA for 12–48 hpi. (B) Quantitative analysis of the number of CFUs of the XC1 strain grown in XYS medium with or without 100 μM IAA. (C) Quantitative analysis of the number of CFUs of the XC1 strain grown in XYS medium supplemented with 0–100 μM IAA at 48 hpi. Three independent experiments were conducted, and data are expressed as the mean ± SD. *p < 0.05; **p < 0.01; ns, not significant.

In XYS medium, the OD_600_ of XC1 culture remained relatively stable at 24–48 hpi (Figure 2A). These cultures were further diluted 10–10^5^ times, and the diluents were plated on XYS agar plates to count the number of colony‐forming units (CFUs). The CFUs of XC1 culture were observed to dramatically decrease after 24 hpi in XYS medium (Figure 6A). Further quantitative analysis indicated that the CFUs of XC1 culture decreased from 1.65 × 10^11^ at 24 hpi to 2.65 × 10^5^ at 48 hpi (Figure 6B). These results indicate that XC1 undergoes cell death in the late growth phases in XYS medium.

To explore the mechanisms underlying XC1 cell death during growth and the IAA‐induced virulence factor production and virulence, the effects of IAA on XC1 CFUs were examined. The addition of IAA had no significant effect on XC1 CFUs at 12–24 hpi (Figure 6A). However, the CFUs of IAA‐treated XC1 were significantly higher than those of nontreated XC1 at 36–48 hpi (Figure 6A). The number of CFUs produced by nontreated XC1 and 100 μM IAA‐treated XC1 at 48 hpi was 2.60 × 10^5^ and 1.39 × 10^10^ CFU/ml, respectively (Figure 6B), representing an increase of 53,461‐fold. Concentration‐dependent effects of IAA on CFUs were observed in XC1 culture (Figure 6C). In contrast, when XC1 was grown in the rich media NA and NYG, the addition of 100 μM IAA had no significant effect on the number of CFUs (Figure S6A,B). This is highly consistent with the previous finding that the phytopathogen Xcc displayed some virulence‐related phenotypes exclusively in the XYS medium, mimicking the in planta conditions during Xcc infection inside host plants^28^. When XC1 grows in XYS medium, it induces an acidic environment, similar to the acidic apoplastic and vascular pH of plants, which may be critical for the expression of virulence factors in XC1^35^.

**Figure 7:**
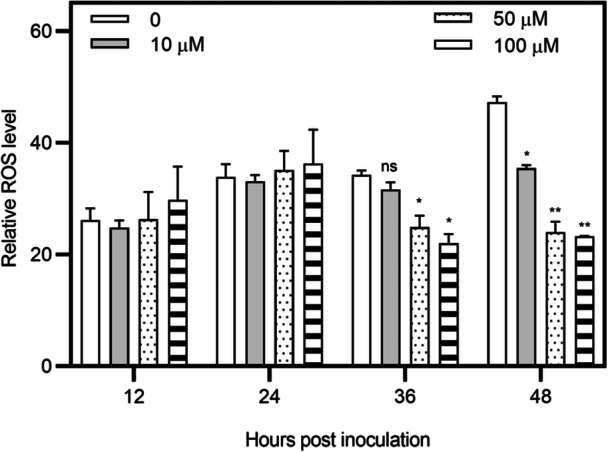
Relative reactive oxygen species (ROS) level of the XC1 strain in XYS medium supplemented with 0–100 μM IAA at 12–48 hpi. Three independent experiments were conducted, and data are expressed as the mean ± SD. *p < 0.05; **p < 0.01; ns, not significant.

To understand the mechanisms underlying IAA‐induced cell viability, the endogenous ROS levels in IAA‐treated XC1 and nontreated XC1 were determined. IAA addition to the XC1 culture had no significant effect on the ROS level at 12–24 hpi; however, it significantly reduced the endogenous ROS level of XC1 at 36–48 hpi (Figure 7). These results suggest that IAA negatively regulates ROS production in XC1 at the stationary phase in XYS medium.

### IAA negatively regulates transcription of the *ilvCGM*‐*leuA* cluster and biosynthesis of BCAAs

**Figure 8:**
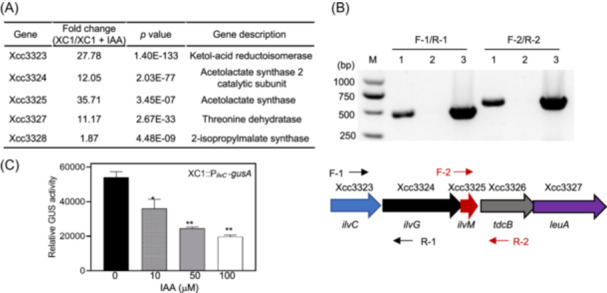
IAA negatively regulates *ilvCGM‐leuA* transcription. (A) RNA‐Seq analysis identified the fold change of the *ilvCGM‐leuA* transcriptional levels in the absence and presence of 100 μM IAA. (B) Reverse‐transcription polymerase chain reaction (RT‐PCR) analysis to confirm the single operon in *ilvCGM‐leuA*. (C) Relative GUS activity in reporter strain XC1::P_*ilvC*_‐*gusA* at 24 hpi in the presence of 10–100 μM IAA. Three independent experiments were conducted, and data are expressed as the mean ± SD. *p ≤ 0.05; **p ≤ 0.01.

To explore the molecular mechanisms underlying IAA regulation in Xcc, RNA sequencing (RNA‐Seq) analysis was conducted to compare the transcriptional differences when Xcc was grown in the presence and absence of 100 μM IAA. The transcriptional level of a gene cluster Xcc3323–Xcc3327 (*ilvCGM‐leuA*) was significantly decreased by 1.87–35.71‐fold in the presence of IAA (Figure 8A). Within this cluster, the genes overlapped by 4 bp or 17 bp or separated by 45 bp or 75 bp (Figure S7). Reverse transcription polymerase chain reaction (RT‐PCR) analysis identified the specific DNA fragment corresponding to the two gap regions (Figure 8B). These results suggest that all five genes within the *ilvCGM‐leuA* cluster were transcribed as an operon, sharing a common promoter located upstream of *ilvC* (Figure 8B). To further confirm the IAA‐repressed expression of *ilvCGM‐leuA*, reporter strain XC1::P_*ilvC*_‐*gusA* was generated by fusing the ∼500 bp of the *ilvC* promoter region with the *gusA* gene (Figure 8C). In the presence of 10–100 μM IAA, the relative GUS activity in the XC1::P_*ilvC*_‐*gusA* strain significantly decreased (33.4%–63.7%) in a concentration‐dependent manner (Figure 8C). These results confirm that the expression of the gene cluster *ilvCGM‐leuA* is repressed by IAA.

**Figure 9:**
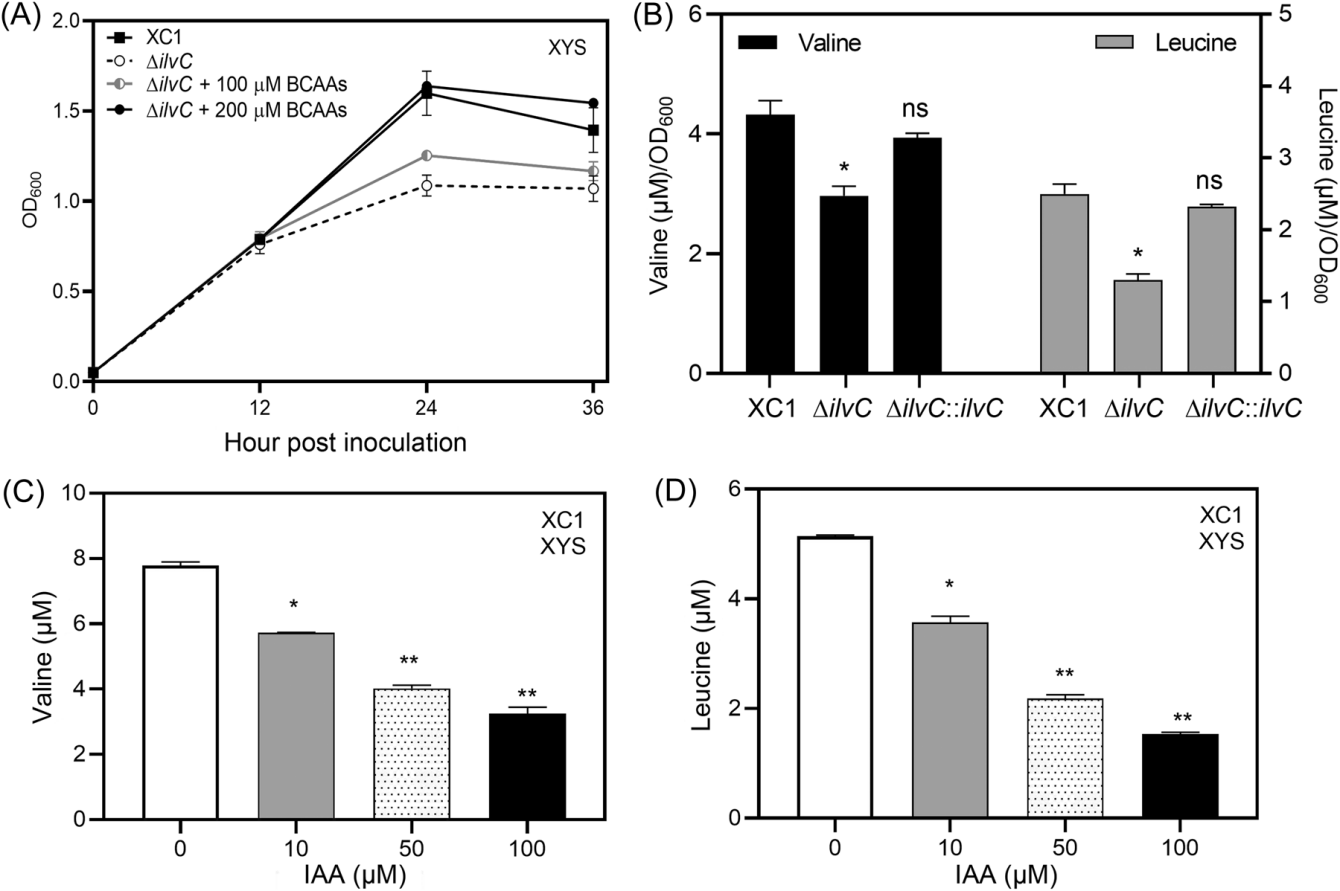
IAA negatively regulates the endogenous levels of valine and leucine in Xcc. (A) Growth of strains XC1 and Δ*ilvC* in the absence and presence of branched‐chain amino acids (BCAAs). (B) Relative endogenous levels of valine and leucine in strains XC1, Δ*ilvC*, and Δ*ilvC*::*ilvC* in XYS. (C, D) Endogenous level of valine (C) and leucine (D) in the presence of 10–100 μM IAA.Three independent experiments were conducted, and data are expressed as the mean ± SD. *p < 0.05; **p < 0.01; ns, not significant.

Xcc3323 encodes an NADP‐dependent ketol‐acid reductoisomerase, while Xcc3324 and Xcc3325 encode the acetolactate synthase 2 catalytic and acetolactate synthase isozyme II small subunits, respectively. Xcc3326 and Xcc3327 encode threonine dehydratase and isopropylmalate synthase, respectively. These genes have been proposed to be involved in the biosynthesis of BCAAs (valine, leucine, and isoleucine) in Xcc^36^. In this study, to confirm the involvement of *ilvCGM‐leuA* in BCAA biosynthesis, *ilvC* deletion mutant strain Δ*ilvC* was generated in Xcc strain XC1. When grown in the XYS medium, the Δ*ilvC* strain displayed slower growth than the wild‐type XC1 strain (Figure 9A). The addition of 100 μM BCAAs (100 μM valine + 100 μM leucine + 100 μM isoleucine) to the Δ*ilvC* strain significantly increased the growth, as indicated by OD_600_, and the addition of 200 μM BCAAs fully restored the growth to the level of the wild‐type (Figure 9A). An HPLC‐based method for quantitative analysis of valine and leucine was established in this study (Figure S8). The endogenous levels of valine and leucine in the Δ*ilvC* strain were significantly lower than those in the wild‐type XC1 strain. Integration of a single copy of *ilvC* in the Δ*ilvC* strain restored the levels of valine and leucine to those of the wild‐type strain (Figure 9B). These results suggest that the gene cluster *ilvCGM‐leuA* is involved in BCAA biosynthesis.

Furthermore, the endogenous valine and leucine concentrations in wild‐type XC1 in the presence of 10–100 μM IAA were determined. IAA addition significantly decreased the endogenous levels of valine and leucine in a concentration‐dependent manner (Figure 9C,D). Taken together, these results suggest that IAA negatively regulates BCAA biosynthesis via the *ilvCGM‐leuA* gene cluster.

### BCAAs are associated with cell death and ROS levels in XC1

**Figure 10:**
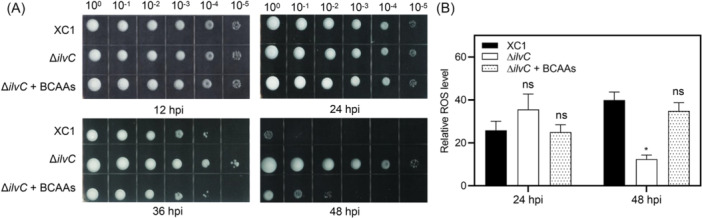
Effects of *ilvC* and BCAAs on cell viability and ROS levels in Xcc. (A) CFUs of strains XC1 and Δ*ilvC* in the absence and presence of 200 μM BCAAs in XYS medium. (B) Relative ROS levels of strains XC1 and Δ*ilvC* in the absence and presence of 200 μM BCAAs. Three independent experiments were conducted, and data are expressed as the mean ± SD. *p < 0.05; ns, not significant.

To determine the role of BCAAs in Xcc cell death, colony formation was analyzed in wild‐type strain XC1 and mutant strain Δ*ilvC*. Deletion of *ilvC* in XC1 showed no significant effect on the number of CFUs at 12 hpi and 24 hpi; however, it significantly prevented cell death at 36 hpi and 48 hpi (Figure 10A). The addition of 200 μM BCAAs to the Δ*ilvC* strain restored the number of CFUs at 36 hpi and 48 hpi to the level of wild‐type XC1 (Figure 10A). These results suggest that BCAAs are positively associated with cell death in XC1.

The role of BCAAs in ROS production was analyzed using strains XC1 and Δ*ilvC*. Deletion of *ilvC* in XC1 had no significant effect on the ROS level at 24 hpi; however, it significantly reduced the ROS level at 48 hpi (Figure 10B). The addition of 200 μM BCAAs to mutant strain Δ*ilvC* had no significant effect on the ROS level at 24 hpi; however, it significantly restored the ROS level to that of the wild‐type strain at 48 hpi (Figure 10B). These results suggest that BCAAs are negatively associated with Xcc survival in stationary‐phase cultures.

## DISCUSSION

Microbial synthesis of IAA mainly occurs through l‐Trp‐dependent pathways^7^. In l‐Trp‐dependent pathways, l‐Trp is converted into various intermediate metabolites. Depending on the bacterial species, these intermediate metabolites are further involved in IAA biosynthesis through the IAM‐, IPA‐, IAN‐, TAM‐, or TSO (l‐Trp side‐chain oxidase)‐dependent sub‐pathways^16^, ^37^, ^38^, ^39^, ^40^ (Figure 4A). In this study, we showed that Xcc strain XC1 produced IAA via an l‐Trp‐dependent pathway. Further bioinformatic analysis identified 15 genes homologous to the previously reported IAA biosynthetic genes in plant‐associated bacteria, plant pathogens, fungi, and rice (Figure S3). Gene deletion and overexpression analysis revealed that the gene Xcc1569 was partially associated with IAA biosynthesis in XC1 (Figures S4 and S5). Furthermore, among the three previously reported intermediates in IAA biosynthesis, namely, TOL, IAM, and IAN, only TOL was shown to partially promote IAA biosynthesis in XC1 (Figure 4B–E). TOL is easily converted into IAAld, which is an important intermediate in the IPA‐, TAM‐, and TSO‐dependent sub‐pathways (Figure 4A)^37^, ^40^. Thus, XC1 seems to have multiple sub‐pathways in the l‐Trp‐dependent IAA biosynthetic pathway, and the Xcc1569‐TOL‐IAAld sub‐pathway probably represents one of them. These results further support the current consensus that IAA production in bacteria is subject to species and strain variations as well as the culture conditions, growth stage, and substrate availability^7^, ^41^. Nevertheless, the genes and complete sub‐pathway for l‐Trp‐dependent IAA biosynthesis in Xcc remain to be elucidated.

The present study also showed that Phe and Tyr can be used by XC1 to produce IAA, although they are less effective than l‐Trp (Figure 3C–G). The mechanisms underlying Phe‐ or Tyr‐dependent IAA biosynthesis deserve further investigation. It is possible that Phe or Tyr is converted into l‐Trp‐derived intermediates in XC1 because l‐Trp, Phe, and Tyr belong to the same aromatic amino acid group. However, we cannot rule out the possibility that Xcc has multiple routes and intricate biosynthetic pathways for IAA biosynthesis, as demonstrated in many rhizosphere bacterial strains, such as *Pseudomonas* sp. UW4^17^. This redundancy proves advantageous, as one bacterial strain failing to produce IAA through one specific IAA biosynthetic pathway can still produce IAA through an alternative route^7^. These results highlight the significance of the ability to produce IAA in the adaptation and virulence of the phytopathogen Xcc.

As a vascular pathogen, Xcc multiplies inside the xylem of crucifer plants by producing multiple degradative enzymes to degrade the whole plant cell via a DSF‐dependent quorum‐sensing mechanism^30^, ^42^, ^43^, ^44^. Simple sugars and amino acids are released from degraded cells to support Xcc growth and colonization^44^. During infection, Xcc scavenge host plant‐derived phenolic acids, l‐Trp, Phe, and Tyr, and convert them into IAA^45^, ^46^ (Figure 3C–G). Moreover, Xcc infection was shown to stimulate the host plant to produce more IAA. The IAA level in the leaf tissue of *Arabidopsis thaliana* increased in response to Xcc infection^31^. Host plant‐derived IAA also acts directly on the invading pathogens. Thus, the invading phytopathogen Xcc is likely to be exposed to high levels of IAA during infection. How the invading Xcc deals with this high IAA level is a fundamental biological question in the molecular interactions between Xcc and host plants. This has not been fully determined and needs to be investigated in the future.

Despite extensive research on the function of IAA in plants, there is still limited knowledge about its functions in microbes, in particular, in phytopathogens^7^. The present study demonstrated that IAA had a global effect on Xcc strain XC1. IAA positively regulated EPS production, extracellular protease activity, and cell viability (Figures 5B,C and 6) and negatively regulated BCAA biosynthesis and ROS production inside the cell (Figures 7 and 9C,D). IAA treatment also promoted the virulence of XC1 in the host plant (Figure 5D,E). Further analysis identified a putative IAA signaling cascade involving *ilvCGM‐leuA* expression, BCAA production, ROS production, and increased colony formation and virulence in Xcc strain XC1. These results reveal the dual roles of IAA in Xcc–plant interactions. IAA likely exerts a regulatory effect on both plant cells and invading pathogens. In return, Xcc has probably evolved a mechanism to sense IAA as a stress signal to promote its survival and virulence during infection inside the host plant. A similar mechanism has recently been reported in the salicylic acid (SA)‐mediated Xcc–plant interaction^35^. Xcc infection induced SA production at the infection site in cabbage. SA not only regulates host immunity but also acts on the invading Xcc to induce turnover of the quorum‐sensing signal, DSF^35^. During cabbage infection, XC1 can sense SA via a transcription factor, HepR, and efflux SA via an RND family efflux system, HepABCD, to promote its virulence^47^. These results describe a plant hormone‐mediated fine‐tuned interaction network between Xcc and host plants.

BCAAs are important nutrients in bacterial physiology. They are not only the blocks for protein synthesis but also signals for fine‐tuning the adaptation to amino acid starvation in Gram‐negative and Gram‐positive bacteria^48^. BCAA depletion has been shown to trigger the synthesis of ppGpp, an alarmone synthesized from GTP during the stringent response induced by amino acid starvation, correlating with entry into the stationary phase^48^. The present study revealed that IAA treatment decreased *ilvCGM*‐*leuA* expression and BCAA production in XC1 (Figures 8C and 9C,D). Moreover, IAA‐induced changes in ROS levels and cell viability were only observed in the stationary phase (36–48 hpi), not in the exponential phase (12–24 hpi) (Figures 6 and 7). How BCAA starvation triggers altered ROS production and cell viability at the stationary phase remains to be explored. BCAAs have been demonstrated to be effectors of the transcriptional regulator leucine‐responsive regulatory protein (Lrp) in Gram‐negative bacteria and CodY in Gram‐positive bacteria^49^, ^50^. Upon nutrient exhaustion, Lrp or CodY regulates metabolic reprogramming to sustain growth, as shown by the characteristic metabolic shift to the stationary phase^48^. We identified the Lrp‐encoding gene in the genomes of Xcc strains 8004, ATCC33913, and B100. Whether the Lrp protein mediates the BCAA regulation of ROS and cell viability in XC1 will be investigated in the future.

**Figure 11:**
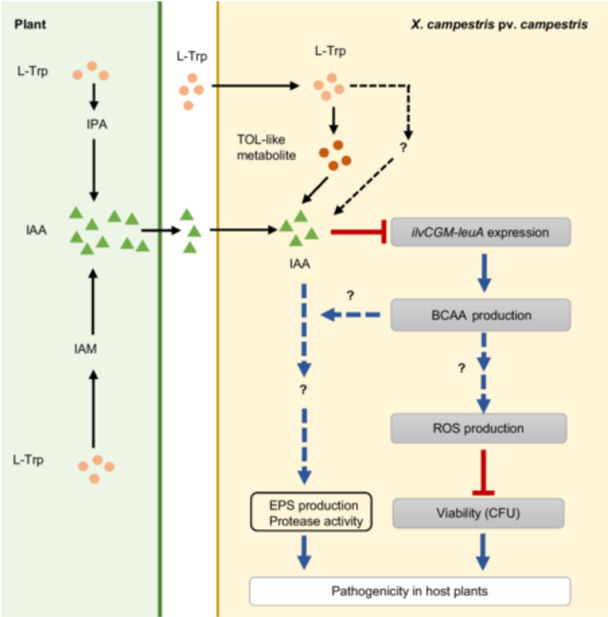
Schematic model for l‐Trp‐dependent IAA biosynthesis and IAA‐mediated interactions between the phytopathogen Xcc and host plants. Xcc utilizes an l‐Trp‐dependent IAA biosynthesis pathway. In this pathway, TOL is one of the intermediates. Xcc can sense and uptake host plant‐derived IAA into the cells. Inside Xcc, IAA negatively regulates transcription of the *ilvCGM*‐*leuA* cluster, resulting in decreased BCAA production. BCAAs positively regulate ROS production, and ROS accumulation leads to decreased CFUs in Xcc. IAA may have an alternative signaling pathway to regulate EPS production and protease activity. These signaling pathways together contribute to the virulence of Xcc in host plants.

Based on our results, we proposed a schematic model that describes how Xcc produces IAA and how IAA regulates the evaluated biological functions in Xcc (Figure 11). Xcc utilizes an l‐Trp‐dependent IAA biosynthesis pathway. In this pathway, l‐Trp is converted into TOL‐like metabolite, which is further used for IAA biosynthesis. It is also possible that l‐Trp is converted into an alternative uncharacterized intermediate for IAA biosynthesis. Xcc senses and uptakes host plant‐derived IAA into the cells. Inside the Xcc cells, IAA negatively regulates transcription of the *ilvCGM*‐*leuA* cluster, resulting in decreased BCAA production in Xcc. BCAAs positively regulate ROS production, and ROS accumulation inside Xcc cells leads to decreased viability. IAA may have an alternative signaling pathway to regulate EPS production and protease activity. These signaling pathways together contribute to the virulence of Xcc in host plants (Figure 11). Future studies will provide more insights into how IAA regulates the expression of the *ilvCGM*‐*leuA* cluster, how BCAAs regulate ROS production, and how IAA regulates EPS production and protease activity.

## MATERIALS AND METHODS

### Bacterial strains and culture conditions

The bacterial strains and plasmids used in the present study are listed in Table S1. Xcc wild‐type strain XC1 and the XC1‐derived mutant strains were cultivated at 28°C on XYS medium (0.7 g l^−1^ K_2_HPO_4_, 0.2 g l^−1^ KH_2_PO_4_, 1 g l^−1^ (NH_4_)_2_SO_4_, 0.1 g l^−1^ MgCl_2_·6H_2_O, 0.01 g l^−1^ FeSO_4_·7H_2_O, 0.001 g l^−1^ MnCl_2_·4H_2_O, 5 g l^−1^ sucrose, and 0.0625% yeast extract, with a pH of 7.0), NYG medium (5 g l^−1^ peptone, 3 g l^−1^ yeast extract, and 20 g l^−1^ glycerol), or NA medium (5 g l^−1^, 3 g l^−1^ beef extract, 10 g l^−1^ sucrose, and 1 g l^−1^ yeast extract, with a pH of 7.0). *Escherichia coli* strains were used as hosts for constructing all recombinant vectors and were cultured on Luria–Bertani (LB) medium (5 g l^−1^ yeast extract, 10 g l^−1^ peptone, and 10 g l^−1^ sodium chloride) at 37°C. Antibiotics rifamycin (Rif; 25 μg ml^−1^) and kanamycin (Km; 50 μg ml^−1^) were added as needed. Bacterial growth was assessed by measuring the optical density at a wavelength of 600 nm (OD_600_).

### HPLC‐based quantitative analysis of IAA levels in XC1 cultures

To quantitatively evaluate the IAA level in XC1 culture, 7 methanol solutions containing 1.0, 2.5, 5.0, 10, 50, 100, and 250 μM commercially available IAA were prepared and submitted for HPLC analysis. The plot between the peak area (A) of the chromatogram and the IAA concentration (*C*_IAA_) was obtained as follows: *A* = 9.184*C_IAA_ + 195.4 (Figure S1).

IAA extraction and quantitative analysis were performed following a previously described method^51^. Briefly, the Xcc strains were cultured in XYS, NYG, or NA media for 12–48 h. A volume of 50 ml of the XC1 culture supernatant was prepared by centrifugation at 10,000 rpm for 30 min at 4°C (HERAEUS MULTIFUGE X1R Centrifuge; Thermo Fisher Scientific Inc.). Hydrochloric acid was added to the supernatants to adjust the pH to 3.0. IAA was extracted with an equal volume of ethyl acetate. The fractions of the ethyl acetate phase were evaporated and dissolved in 0.1 ml of methanol for HPLC analysis. HPLC analysis was conducted using a C18 reverse‐phase column (Zorbax XDB; 5 μm, 4.6 × 150 mm; Agilent). The chromatographic separation was performed using a gradient elution program initiated with methanol in water (10:90, v:v, 0.1% acetic acid). A linear gradient ramp was then implemented over 40 min to achieve a methanol concentration of 35% (v/v, 0.1% acetic acid). Following gradient completion, the final mobile phase composition was sustained for 10 min for the next run. All separations were conducted at a constant flow rate of 0.8 ml/min.

### Mass spectrometry to verify the identity of IAA

IAA was extracted and purified following the method previously described by Shao et al.^51^. Confirmation of the identity of IAA was performed using UPLC‐TOF‐MS. Briefly, 50 ml of liquid culture was harvested from each experimental strain. Subsequently, the cultures were acidified to pH 3.0 using hydrochloric acid, followed by liquid–liquid extraction with an equal volume (50 ml) of ethyl acetate. Ethyl acetate was subsequently eliminated through rotary evaporation conducted at a temperature of 30°C. Following this step, the residues were dissolved in 0.1 ml of methanol and subjected to analysis using UPLC‐TOF MS (Agilent) with a C18 reverse‐phase column (Zorbax XDB; 5 mm, 4.6 × 150 mm; Agilent, Santa Clara). The sample was initially eluted using a solution of water and methanol (70:30, v:v) for 2 min at a rate of 0.4 ml/min. A gradual increase in methanol to 60% was initiated over the course of 10 min.

### Bioinformatics analysis to identify the putative genes involved in IAA biosynthesis

The amino acid sequences of all previously described genes involved in l‐Trp conversion into IAA were downloaded from NCBI. BlastP and domain organization analyses were conducted using the SMART program (http://smart.embl-heidelberg.de/) to identify homologous genes in Xcc. These genes were further used for genetic analysis of their roles in IAA biosynthesis.

### Gene deletion, overexpression, and complementation analysis

Gene deletion, overexpression, and complementation were performed following the protocol previously described by He et al.^42^. Briefly, the fusion DNA fragments containing ~500 bp upstream and ~500 bp downstream regions of the target gene were cloned into the suicide vector pK18mobsacB using a ClonExpress MultiS one‐step cloning kit (Vazyme Biotech Co., Ltd.). The constructed recombinant plasmid was then introduced into XC1 through mating. The resultant Km‐resistant colony was then plated on NA plates with 25 μg ml^−1^ Rif and 5% (w:v) sucrose for 48 h. The in‐frame gene deletion strain was finally screened using PCR and subsequent DNA sequencing. The primers used in this process are listed in Table S1.

To generate a gene overexpression strain, the target gene was amplified by PCR and cloned into the multiple cloning site of the pBBR‐1‐MCS2 expression plasmid. The resulting recombinant plasmids were introduced into XC1 strains by triparental mating. The primers used are detailed in Table S2.

To perform single‐copy complementation of *ilvC*, the DNA fragment containing the *ilvC* coding region along with its 542 bp upstream of the translational start codon was PCR‐amplified and cloned into delivery vector mini‐Tn7T‐Gm. The resulting constructs were electroporated into strain Δ*ilvC*, following the methodology previously described by Jittawuttipoka et al.^52^.

### Quantitative determination of EPS production and extracellular protease activity

EPS production and extracellular enzyme activity were quantitatively determined following the methods previously described by He et al.^42^. Briefly, 15 ml of XYS cultures were collected at 24 and 36 hpi and centrifuged at 12,000 rpm for 30 min to prepare the supernatant. The supernatant was then mixed with 2 volumes of absolute ethanol and kept at −20°C for 20 min. The precipitated EPS was spun down and dried in a 55°C oven overnight before determining the dry weight.

The extracellular protease activity of the XC1 culture was measured using the azocasein assay described by He et al.^42^. Briefly, azocasein (Sigma‐Aldrich, Co.) was dissolved in 0.1 M Tris‐HCl (pH 8.0) as a substrate solution. Then, 100 μl of the supernatant of the XC1 XYS culture was collected at 48 hpi and mixed with 200 μl of the substrate solution. After incubating at 37°C for 3 h, samples were mixed with 300 μl of ice‐cold 10% trichloroacetic acid solution and left on ice for 15 min. The reaction mixture was centrifuged at 12,000 rpm for 10 min, and the optical density of the resultant supernatant was measured at OD_366_.

### Virulence assay of Xcc strains on cabbage

Cabbage (*Brassica oleracea*) cultivar “Jingfeng‐1” (China Vegetable Seed Technology Co., Ltd.) was grown in a growth chamber (Yanghui RDN‐500B) at 28°C and 95% relative humidity with a photoperiod regime of 16 h light (8000 Lx). The virulence assay was conducted using the leaf‐clipping method described by Chen et al.^45^. Briefly, the Xcc strains were grown in an XYS liquid medium with or without 100 μM IAA for 12 h. The collected cell pellets were resuspended in 1× phosphate‐buffered saline (PBS) to a final OD_600_ of 0.1. For each strain, 12 2‐month‐old cabbage leaves were inoculated, and the lesion lengths were quantified at 12 dpi. Mutant strain Δ*rpfC* was used as a negative control for virulence evaluation in this study.

### Colony formation of Xcc strains

The Xcc strains were grown in XYS liquid medium supplemented with 0, 10, 50, and 100 μM IAA for 12–48 h, and 1 ml of the culture was collected at 12, 24, 36, and 48 hpi and centrifuged at 8000*g* for 5 min. The resultant cell pellet was resuspended in 1 ml of PBS, washed twice with an equal volume of PBS, and further diluted 10^0^, 10^1^, 10^2^, 10^3^, 10^4^, and 10^5^ times with PBS. A 2 μl sample of each diluent was plated onto an XYS agar plate supplemented with 25 μg/ml rifampicin and incubated for 48 h for colony formation.

To quantitatively determine the CFUs of Xcc strains, a 100‐μl aliquot of the diluted PBS cultures was plated onto XYS agar plates supplemented with 25 μg/ml rifampicin. The visible CFUs were recorded for each treatment at 48 hpi.

### Quantification of the ROS content

The ROS content was determined using a commercially available ROS Assay Kit (Beyotime Biotechnology). Briefly, the Xcc strains were grown in XYS liquid medium supplemented with 0, 10, 50, and 100 μM IAA for 24–48 h. Then, 300 μl of the Xcc cell cultures were collected and centrifuged at 8000*g* for 5 min at 4°C. The resultant cell pellets were washed twice with PBS and resuspended in 600 μl of PBS containing 10 μM 2′,7′‐dichlorodihydrofluorescein diacetate (DCF‐DA) for 30 min at 28°C in the dark. The treated bacterial cells were transferred into the wells of a multi‐mode microplate reader (SpectraMax M5; Molecular Devices). The fluorescent value of each well was read at excitation and emission wavelengths of 488 nm and 525 nm, respectively.

### RT‐PCR analysis

XC1 cells were collected from the XYS culture at 12 hpi. The total RNA of XC1 was isolated and purified using a RNeasy Miniprep Kit (Qiagen). A genomic DNA (gDNA) eraser was used to remove the residual gDNA. The complementary DNA (cDNA) synthesis was conducted using the PrimeScript RT Reagent Kit (Takara Bio Inc.). The primers used in this study are detailed in Table S2.

### RNA‐Seq analysis

XC1 strain was cultured in 50 ml of liquid XYS medium supplemented with 0 or 100 μM IAA for 24 h at 28°C. Bacterial cells were collected by centrifugation at 12,000 rpm for 10 min at 4°C. RNA‐Seq was subsequently performed by Shanghai Personal Biotechnology Co. Ltd. using the Illumina HiSeq system as described by Song et al.^35^.

### Generation of *gusA*‐fusion reporter strains and the GUS activity assay

*gusA*‐fusion reporter strains were constructed for *ilvCGM* following the methodology previously described by Zhou et al.^53^. The DNA fusion fragments, comprising the T0T1 terminator, the promoter region, and the *gusA* gene, were subsequently cloned into the pMini‐Tn7T‐Gm plasmid, thereby generating the mini‐P_*ilvC*_‐*gusA* plasmid. The mini‐P_*ilvC*_‐*gusA* plasmid was integrated into strain XC1 by electroporation, as previously described. Quantitative β‐glucuronidase (GUS) activity assays were conducted following the methodology outlined by Chen et al.^45^. Briefly, the reporter strain XC1::P_*ilvC*_‐*gusA* was cultivated in XYS medium with 0, 10, 50, and 100 μM IAA for 12–36 h at 28°C. Subsequently, 500‐μl culture samples were collected, centrifuged at 12,000 rpm for 10 min, and washed once with PBS. Subsequently, the bacterial cells were suspended in 300 μl of lysis buffer (TieChui; ACE Biotechnology), subjected to pyrolysis at 4°C for 30 min, and centrifuged at 8000 rpm (ERAEUS MULTIFUGE X1R Centrifuge; Thermo Fisher Scientific Inc.) for 5 min. A 10‐μl sample of the supernatant was collected and added to 250 ml of MUG solution (1 mM 4‐methylumbelliferyl β‐d‐glucuronide, 50 mM PBS, 5 mM DTT, and 1 mM EDTA, pH 8.0). The reaction mixture was incubated at 37°C for 5 min. Each reaction was terminated by adding 800 μl of a 0.4 M Na_2_CO_3_ solution. A 96‐well plate was used to quantify the GUS activity in a multi‐mode microplate reader (SpectraMax M5; Molecular Devices), with excitation and emission wavelengths of 365 and 455 nm, respectively.

### Determination of the branched amino acid concentration

XC1 and mutant strains were cultivated in XYS medium with 0, 10, 50, and 100 μM IAA for 12–24 h at 28°C. A culture sample (10 ml) was collected, centrifuged at 10,000 rpm for 40 min, and washed once with PBS. The bacterial cells were suspended in 400 μl of lysis buffer (TieChui; ACE Biotechnology) and subjected to pyrolysis at 4°C for 30 min. Subsequently, 100 μl of the resulting bacteria lysate was mixed with 100 μl of boric acid buffer (40 mM Na₂B₄O₇ and 40 mM H₃BO₃, pH 9.0) and 100 μl of 1% 2,4‐dinitrofluorobenzene (acetonitrile) incubated in a 60°C water bath in the dark for 60 min. After completing the reaction, 700 μl of phosphate buffer (25 mM KH_2_PO_4_, 14.55 mM NaOH, pH 7.0) was added to the system. Samples were left to stand at room temperature for 15 min and centrifuged at 12,000 rpm for 10 min. The supernatants were subjected to HPLC analysis using a C18 reverse‐phase column (Zorbax XDB; 5 mm, 4.6 × 150 mm). The mobile phase comprised 50 mM sodium acetate (1% DMF, pH 7.2) (solvent A) and 50% acetonitrile (solvent B) at a flow rate of 1.2 ml min^−1^.

### Statistical analyses

All experiments were replicated in triplicate unless specified otherwise. An analysis of variance (ANOVA) was conducted for the experimental datasets implemented in JMP software (version 5.0; SAS Institute Inc.). Significant treatment effects were determined by the *F* value (*p *< 0.05).

## AUTHOR CONTRIBUTIONS

**Sinan Li**: Conceptualization; data curation; formal analysis; methodology; visualization; writing—original draft; writing—review and editing. **Kai Song**: Conceptualization; data curation; formal analysis; methodology; visualization. **Ying Cui**: Data curation; formal analysis; methodology; visualization. **Lin Li**: Data curation; formal analysis; methodology. **Minglei Zhang**: Data curation; formal analysis; methodology. **Ya‐Wen He**: Conceptualization; data curation; formal analysis; funding acquisition; project administration; writing—original draft; writing—review and editing.

## ETHICS STATEMENT

This study did not involve human subjects and animals.

## CONFLICT OF INTERESTS

The authors declare no conflict of interests.

## Supporting information

Supporting information for mLife resubmission4.

## Data Availability

The authors confirm that the data supporting the findings of this study are available within the article and its supporting information materials.
