# Supplementary material for: Xanthomonas campestris utilizes IAA to regulate its viability and virulence by altering the production of BCAAs and ROS
Source: mLife. 2025 Oct 27;4(5):551–66. doi: 10.1002/mlf2.70033 (PMC12575087; doi:10.1002/mlf2.70033)
Supplement: Supplementary file 1 — Supporting information for mLife resubmission4. [file MLF2-4-551-s001.pdf]

A

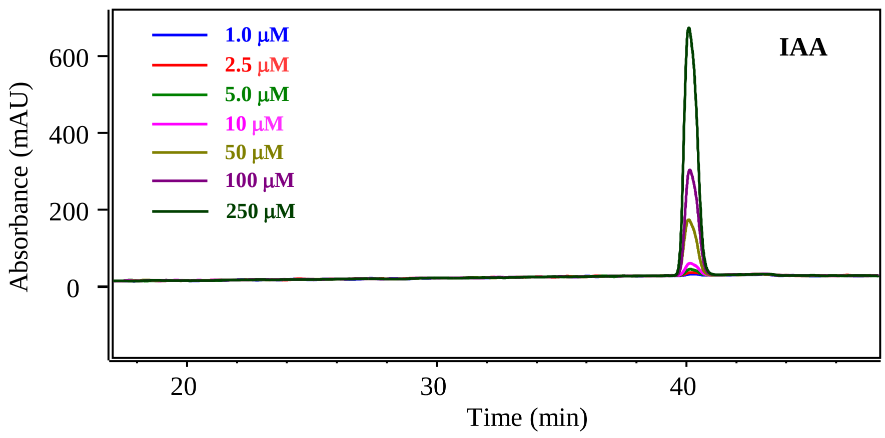

B

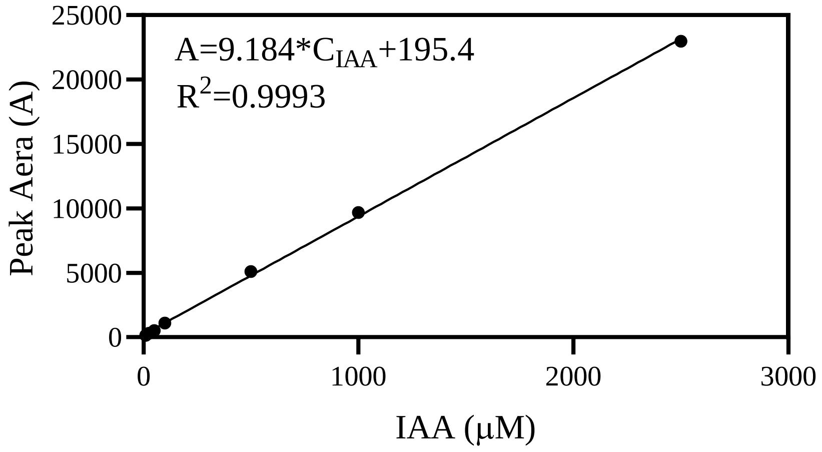

Figure S1. Establishment of a single standard curve for quantification of IAA concentration (C_{IAA}) using the peak aera (A) derived from high performance liquid chromatography (HPLC) analysis. (A) HPLC spectra of IAA at 10 μM to 250 μM; (B) The plot between the peak area (A) of the chromatogram and IAA concentration (C_{IAA}).

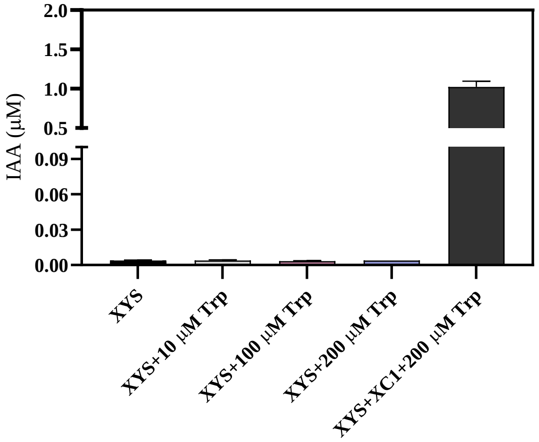

Figure S2. Trp was only converted into IAA in XYS medium with XC1, but not in XYS medium. The level of IAA was measured at 12 hpi. Three independent experiments were conducted and averages along with standard deviations are shown.

Figure S3. Identification of the putative genes involved in IAA biosynthesis in Xcc by BlastP analysis and domain organization analysis using the SMART program (http://smart.embl-heidelberg.de/). IPA: indole-3-pyruvic acid pathway; TSO: tryptophan side-chain oxidase pathway; TAM: tryptamine pathway; IAM: indole-3-acetamide pathway; IAN: indole-3-acetonitrile.

**Pathways and enzymes**	**The reported IAA synthetic genes in microbes** (gene, bacterial species, enzyme size and domain organization)	**The putative homologous gene in Xcc** (gene, enzyme size, domain organization and and identity)	**Predicted function**	**Reference**
IPA pathway: Aromatic AA aminotransferase	*hisC1*, *Azospirillum brasilense* Sp7, 361 aa 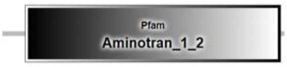	Xcc1569, 420 aa, 27% 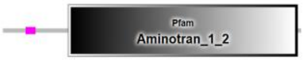 Xcc3275, 399 aa, 30% 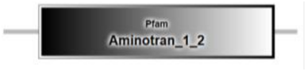	Xcc1569: pyridoxal phosphate-dependent aminotransferase Xcc3275: pyridoxal phosphate-dependent aminotransferase	[1]
	SsAro8, *Sporisorium scitamineum,* 503 aa 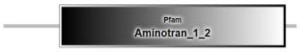	Xcc1838, 410 aa, 27% 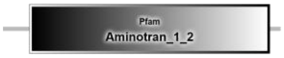	Xcc1838: PLP-dependent aminotransferase family protein	[2]
	MoTam1, *Magnaporthe oryzae, 413 aa* 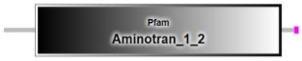	Xcc1838, 410 aa, 27% 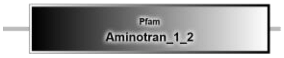	Xcc1838: PLP-dependent aminotransferase family protein	[3]
	TAA1, *Oryza sativa* ssp, 507 aa 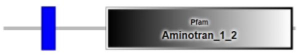	Xcc3275, 399 aa, 32% 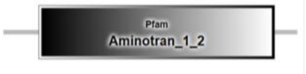	Xcc3275: pyridoxal phosphate-dependent aminotransferase	[4]
IPA pathway: Indole pyruvate decarboxylase	*ipdC*, *Paenibacillus polymyxa* E68, 581 aa 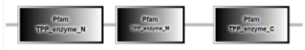	Xcc3324, 573 aa, 48% 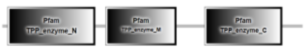	Xcc3324: acetolactate synthase 2 catalytic subunit	[5]
	ipdC, *Enterobacter cloacae*, 550 aa 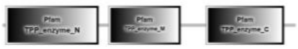	Xcc0206, 575 aa, 25% 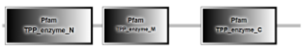	Xcc0206: ubiquinone-depend -ent pyruvate dehydrogenase	[6]
	MoIpd1, *M. oryzae*, 609 aa 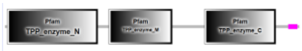	Xcc3324, 573 aa, 25% 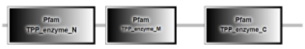	Xcc3324: acetolactate synthase 2 catalytic subunit	[3]
	ipdC, *Bacillus thuringiensis*, 561 aa 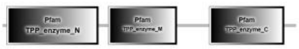	Xcc3324, 573 aa, 26% 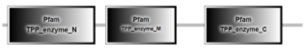	Xcc3324: acetolactate synthase 2 catalytic subunit	[7]

**Pathways and enzymes**	**The reported IAA synthetic genes in microbes** (gene, bacterial species, enzyme size and domain organization)	**The putative homologous gene in Xcc** (gene, enzyme size, domain organization and and identity)	**Predicted function**	**Reference**
IPA pathway: Aldehyde dehydrogenase	AldA, *Pseudomonas syringae* strain DC3000, 497 aa<br>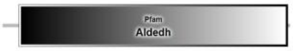 *dhaS*, *B. amyloliquefaciens* SQR9, 495 aa<br>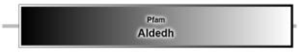 *iad1*, *Ustilago maydis*, 497 aa<br>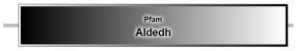	Xcc0101, 509 aa, 41%<br>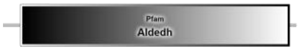 Xcc3403, 490 aa, 41%<br>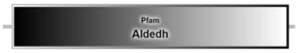 Xcc0354, 503 aa, 34%<br>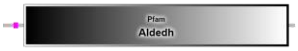 Xcc2336, 454 aa, 30%<br>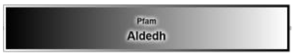 Xcc1260, 501aa, 34%<br>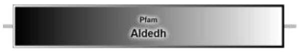 Xcc1791, 510 aa, 29%<br>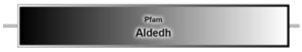	Xcc0101: aldehyde dehydrogenase family protein<br>Xcc3403: betaine-aldehyde dehydrogenase<br>Xcc0354: benzaldehyde dehydrogenase<br>Xcc2336: NAD-dependent succinate-semialdehyde dehydrogenase<br>Xcc1260: CoA-acylating methylmalonate-semialdehyde dehydrogenase<br>Xcc1791: aldehyde dehydrogenase family protein	[8-10]
TAM pathway: Decarboxylase	RUMGNA_01526, *Ruminococcus gnavus*, 490 aa<br>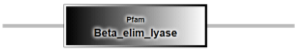	nd	nd	[11]
	tdc, *Rhizobium tropici*, 572 aa<br>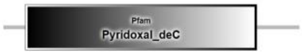	nd	nd	[12]
	MrTDC, *Metarhizium robertsii*, 499 aa<br>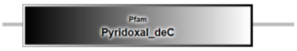	nd	nd	[13]
TAM pathway: Amine oxidase	*aoc1*, *Aspergillus niger*, 671 aa<br>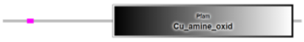	nd	nd	[14]
TSO pathway: Tryptophan side chain oxidase	nd	nd	nd	[15]
IAM pathway: Tryptophan 2-monogoxygenase	*iaaM*, *Erwinia chrysanthemi*, 571 aa<br>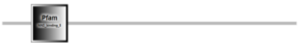	nd	nd	[16]
	iaaM, *P. savastanoi*, 557 aa<br>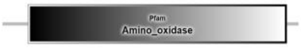	nd	nd	[17]
	iaaM, *Burkholderia pyrrocinia*, 565 aa<br>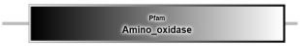	nd	nd	[18]

Pathways and enzymes	The reported IAA synthetic genes in microbes (gene, bacterial species, enzyme size and domain organization)	The putative homologous gene in Xcc (gene, enzyme size, domain organization and and identity)	Predicted function	Reference
IAM pathway: Indole acetamide hydrolase	*iaaH*, *E. chrysanthemi*, 446 aa<br>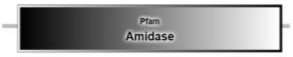<br>*iaaH*, *B. pyrrocinia*, 455 aa<br>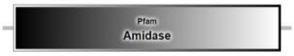	Xcc0924, 505 aa, 40%<br>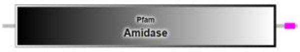<br>Xcc0292, 486 aa, 30%<br>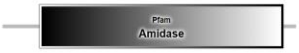	Xcc0924: amidase<br>Xcc0292: AtzE family amidohydrolase	[16, 18]
	IAAMH1, *Arabidopsis thaliana*, 452 aa<br>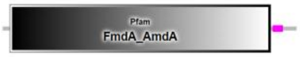	nd	nd	[19]
IAN pathway: Nitrile hydratase	NHase_alpha, *Rhodococcus hodochrous*, 206 aa<br>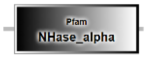	nd	nd	[20]
	NthA, *Pseudomonas* sp. UW4, 199 aa<br>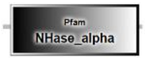	nd	nd	[21]
IAN pathway: Aldoxime dehydratase	OxdRG, *R. globerulus*, 353 aa<br>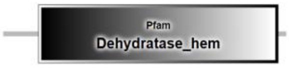	nd	nd	[22]
	OxdK, *Pseudomonas* sp. K-9, 352 aa<br>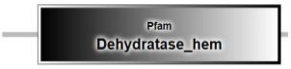	nd	nd	[23]
IAN pathway: Nitrilase	NIT24, *X. oryzae pv.oryzicola*, 294 aa<br>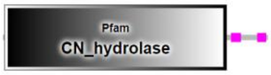	Xcc2688, 294 aa, 86%<br>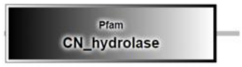	Xcc2688: carbon-nitrogen hydrolase family protein	[24]
	NIT29, *X. oryzae pv.oryzicola*, 267 aa<br>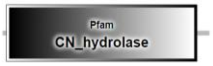	Xcc2217, 266 aa, 90%<br>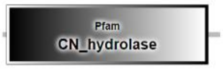	Xcc2217: amidohydrolase	[24]
	Psyr_0007, *P. syringae pv. syringae*, 336 aa<br>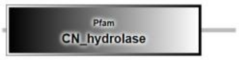	nd	nd	[25]
	Nit, *Pseudomonas* sp. UW4, 307 aa<br>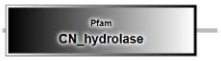	nd	nd	[21]

References

1 Castro-Guerrero J, Romero A, Aguilar JJ, Xiqui ML, Sandoval JO, Baca BE. The *hisC1* gene, encoding aromatic amino acid aminotransferase-1 in *Azospirillum brasilense* Sp7, expressed in wheat. *Plant and soil*. 2012; **356**: 139-150

2 Cui G, Huang C, Bi X, Wang Y, Yin K, Zhu L, et al. Aminotransferase SsAro8 regulates tryptophan metabolism essential for filamentous growth of sugarcane smut fungus *Sporisorium scitamineum*. *Microbiology Spectrum*. 2022; **10**: e00570-00522

3 Dong L, Ma Y, Chen CY, Shen L, Sun W, Cui G, et al. Identification and characterization of auxin/IAA biosynthesis pathway in the rice blast fungus *Magnaporthe oryzae*. *Journal of Fungi*. 2022; **8**: 208

4 Zhang T, Li R, Xing J, Yan L, Wang R, Zhao Y. The YUCCA-auxin-WOX11 module controls crown root development in rice. *Frontiers in Plant Science*. 2018; **9**: 523

5 Phi QT, Park YM, Ryu CM, Park SH, Ghim SY. Functional identification and expression of indole-3-pyruvate decarboxylase from *Paenibacillus polymyxa* E681. *Journal of microbiology and biotechnology*. 2008; **18**: 1235-1244

6 Ryu RJ and Patten CL. Aromatic amino acid-dependent expression of indole-3-pyruvate decarboxylase is regulated by TyrR in *Enterobacter cloacae* UW5. *Journal of bacteriology*. 2008; **190**: 7200-7208

7 Figueredo EF, da Cruz TA, de Almeida JR, Batista BD, Marcon J, de Andrade PAM, et al. The key role of indole-3-acetic acid biosynthesis by *Bacillus thuringiensis* RZ2MS9 in promoting maize growth revealed by the *ipdC* gene knockout mediated by the CRISPR-Cas9 system. *Microbiological Research*. 2023; **266**: 127218

8 McClerklin SA, Lee SG, Harper CP, Nwumeh R, Jez JM, Kunkel BN. Indole-3-acetaldehyde dehydrogenase-dependent auxin synthesis contributes to virulence of *Pseudomonas syringae* strain DC3000. *PLoS pathogens*. 2018; **14**: e1006811

9 Shao J, Li S, Zhang N, Cui X, Zhou X, Zhang G, et al. Analysis and cloning of the synthetic pathway of the phytohormone indole-3-acetic acid in the plant-beneficial *Bacillus amyloliquefaciens* SQR9. *Microbial cell factories*. 2015; **14**: 1-13

10 Reineke G, Heinze B, Schirawski J, Buettner H, Kahmann R, Basse CW. Indole-3-acetic acid (IAA) biosynthesis in the smut fungus *Ustilago maydis* and its relevance for increased IAA levels in infected tissue and host tumour formation. *Molecular plant pathology*. 2008; **9**: 339-355

11 Williams BB, Van Benschoten AH, Cimermancic P, Donia MS, Zimmermann M, Taketani M, et al. Discovery and characterization of gut microbiota decarboxylases that can produce the neurotransmitter tryptamine. *Cell host & microbe*. 2014; **16**: 495-503

12 Imada EL, de Oliveira ALM, Hungria M, Rodrigues EP. Indole-3-acetic acid production via the indole-3-pyruvate pathway by plant growth promoter *Rhizobium tropici* CIAT 899 is strongly inhibited by ammonium. *Research in Microbiology*. 2017; **168**: 283-292

13 Liao X, Lovett B, Fang W, St Leger RJ. *Metarhizium robertsii* produces indole-3-acetic acid, which promotes root growth in *Arabidopsis* and enhances virulence to insects. *Microbiology*. 2017; **163**: 980-991

14 Ai Y, Wang B, Xiao S, Luo S, Wang Y. Tryptophan side-chain oxidase enzyme suppresses hepatocellular carcinoma growth through degradation of tryptophan. *International Journal of Molecular Sciences*. 2021; **22**: 12428

15 Oberhänsli T, Défago G, Haas D. Indole-3-acetic acid (IAA) synthesis in the biocontrol strain CHA0 of *Pseudomonas fluorescens*: role of tryptophan side chain oxidase. *Microbiology*. 1991; **137**: 2273-2279

16 Yang S, Zhang Q, Guo J, Charkowski AO, Glick BR, Ibekwe AM, et al. Global effect of indole-3-acetic acid biosynthesis on multiple virulence factors of *Erwinia chrysanthemi* 3937. *Applied and environmental microbiology*. 2007; **73**: 1079-1088

17 Gaweska HM, Taylor AB, Hart PJ, Fitzpatrick PF. Structure of the flavoprotein tryptophan 2-monooxygenase, a key enzyme in the formation of galls in plants. *Biochemistry*. 2013; **52**: 2620-2626

18 Liu WH, Chen FF, Wang CE, Fu HH, Fang XQ, Ye JR, et al. Indole-3-acetic acid in *Burkholderia pyrrocinia* JK-SH007: Enzymatic identification of the indole-3-acetamide synthesis pathway. *Frontiers in Microbiology*. 2019; **10**: 2559

19 Gao Y, Dai X, Aoi Y, Takebayashi Y, Yang L, Guo X, et al. Two homologous *INDOLE-3-ACETAMIDE (IAM) HYDROLASE* genes are required for the auxin effects of IAM in *Arabidopsis*. *Journal of genetics and genomics*. 2020; **47**: 157-165

20 Komeda H, Kobayashi M, Shimizu S. Characterization of the gene cluster of high-molecular-mass nitrile hydratase (H-NHase) induced by its reaction product in *Rhodococcus rhodochrous* J1. *Proceedings of the National Academy of Sciences*. 1996; **93**: 4267-4272

21 Duca D, Rose DR, Glick BR. Characterization of a nitrilase and a nitrile hydratase from *Pseudomonas* sp. strain UW4 that converts indole-3-acetonitrile to indole-3-acetic acid. *Applied and environmental microbiology*. 2014; **80**: 4640-4649

22 Xie SX, Kato Y, Komeda H, Yoshida S, Asano Y. A gene cluster responsible for alkylaldoxime metabolism coexisting with nitrile hydratase and amidase in *Rhodococcus globerulus* A-4. *Biochemistry*. 2003; **42**: 12056-12066

23 Kato Y and Asano Y. Molecular and enzymatic analysis of the “aldoxime–nitrile pathway” in the glutaronitrile degrader *Pseudomonas* sp. K-9. *Applied microbiology and biotechnology*. 2006; **70**: 92-101

24 Zhang H, Rong Z, Li Y, Yin Z, Lu C, Zhao H, et al. NIT24 and NIT29-mediated IAA synthesis of *Xanthomonas oryzae* pv. *oryzicola* suppresses immunity and boosts growth in rice. *Molecular Plant Pathology*. 2024; **25**: e13409

25 Howden AJ, Rico A, Mentlak T, Miguet L, Preston GM. *Pseudomonas syringae* pv. *syringae* B728a hydrolyses indole-3-acetonitrile to the plant hormone indole-3-acetic acid. *Molecular plant pathology*. 2009; **10**: 857-865

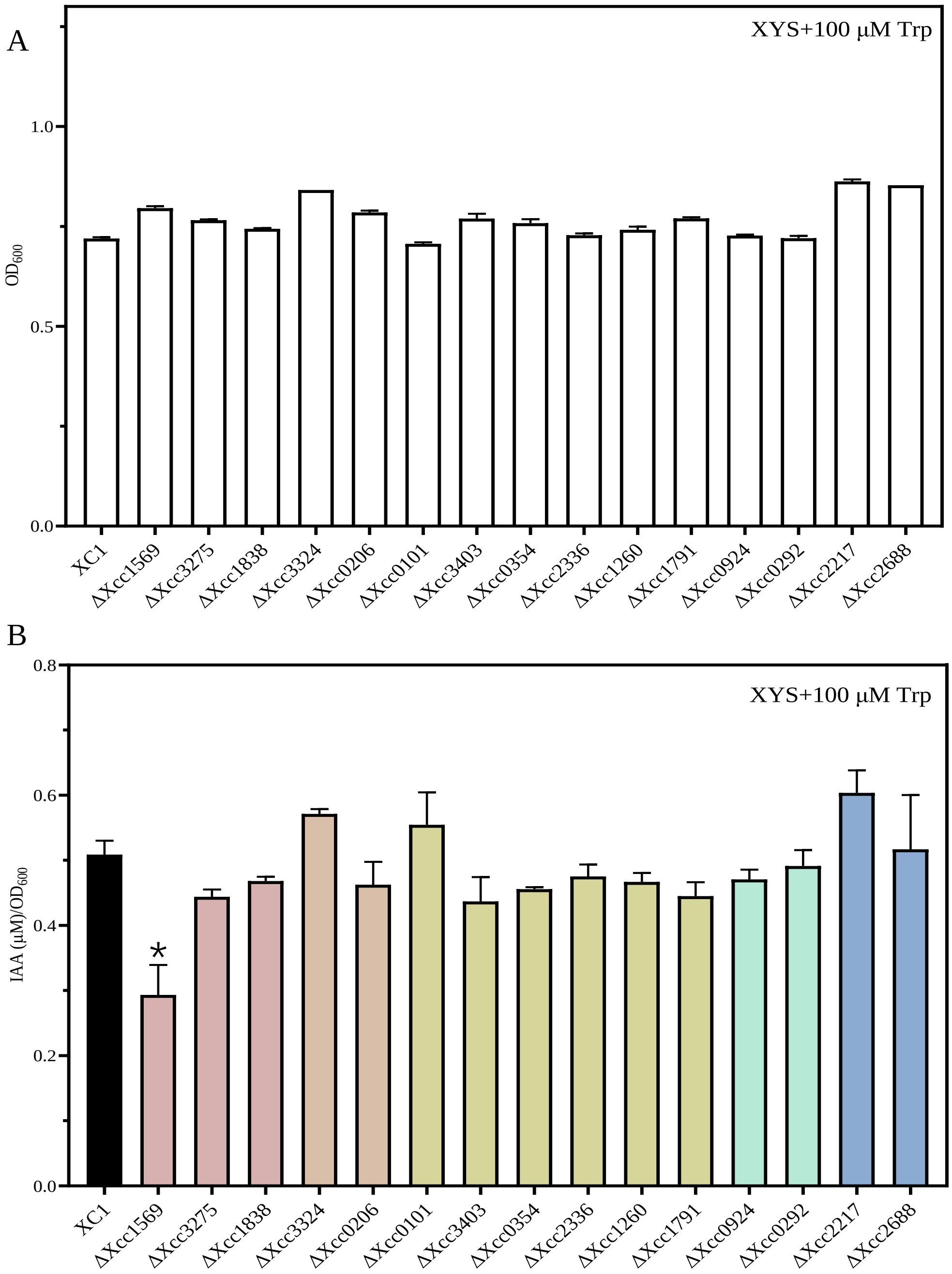

Figure S4. The roles of Xcc genes in IAA biosynthesis. (A) The OD_{600} of the XYS cultures of XC1 and XC1-derived mutant strains at 12 hpi. (B) IAA level of the XC1 strains with single mutation in the putative IAA biosynthetic genes in XYS culture supplemented with 100 μM Trp at 12 hpi. Three independent experiments were conducted and averages along with standard deviations are shown. Statistically significant differences are denoted by one asterisk ($p \leq 0.05$) .

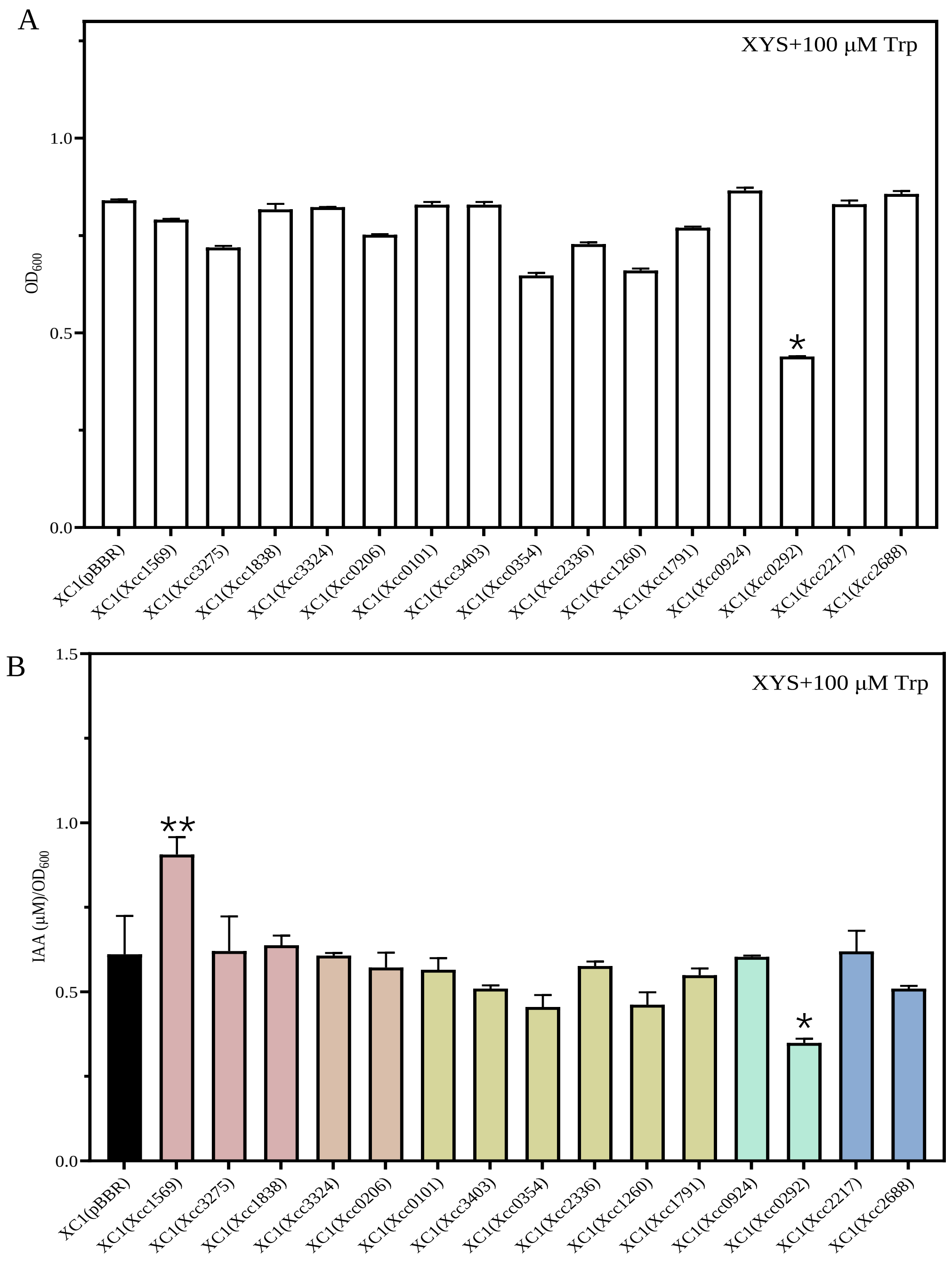

Figure S5. Overexpression analysis to study the roles of the *Xcc* genes in IAA biosynthesis. (A) The OD_{600} of the XYS cultures of XC1 and XC1-derived mutant strains at 12 hpi. (B) IAA level of the XC1 strains overexpressing putative IAA biosynthetic genes in XYS culture supplemented with 100 μM Trp at 12 hpi. Three independent experiments were conducted and averages along with standard deviations are shown. Statistically significant differences are denoted by one asterisk ($p \leq 0.05$) or two asterisks ($p \leq 0.01$) .

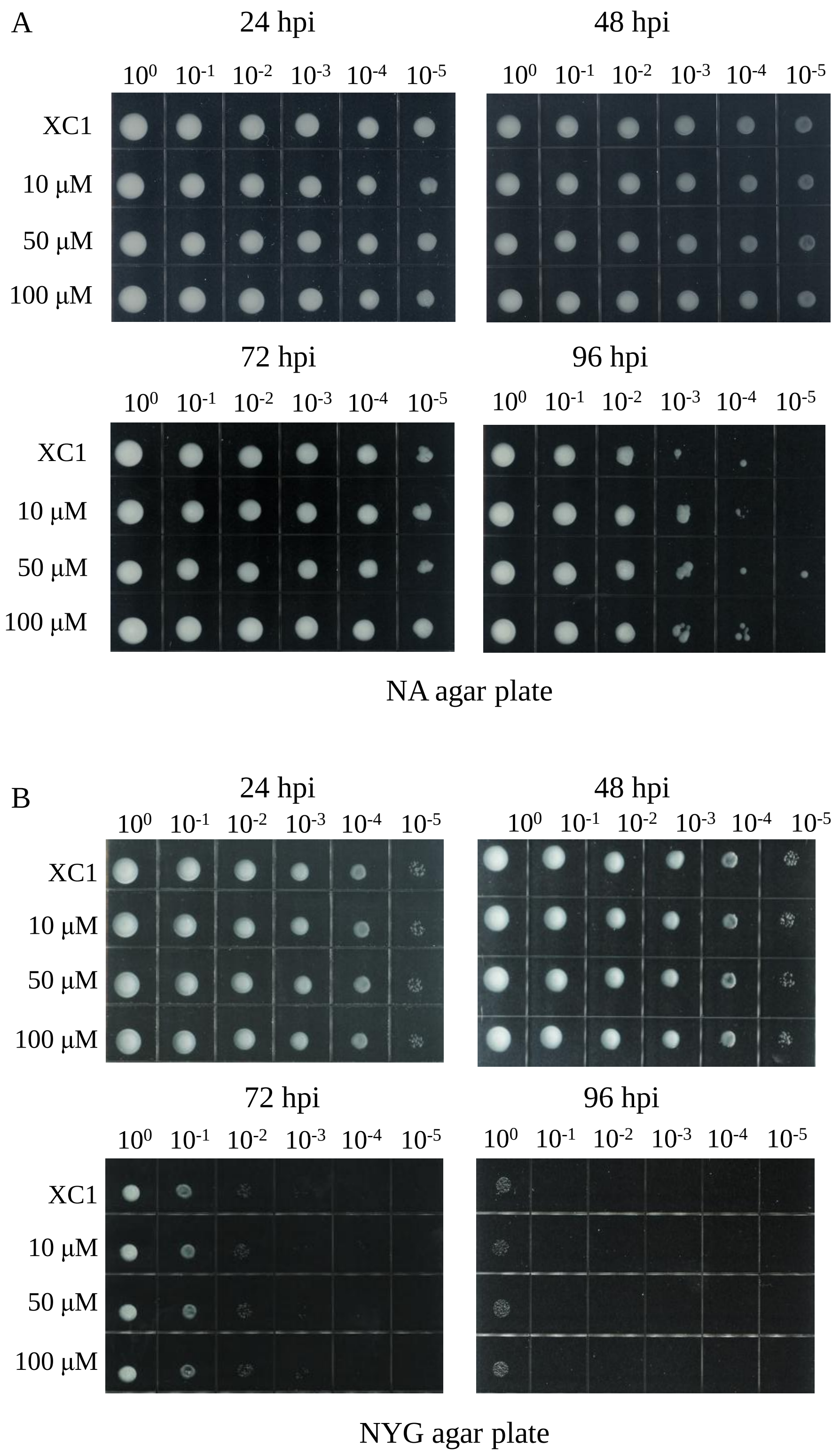

Figure S6. IAA failed to induce an increase in the viability of XC1 in NA and NYG agar plates. (A) CFU on NA agar plate. (B) CFU on NYG agar plate.

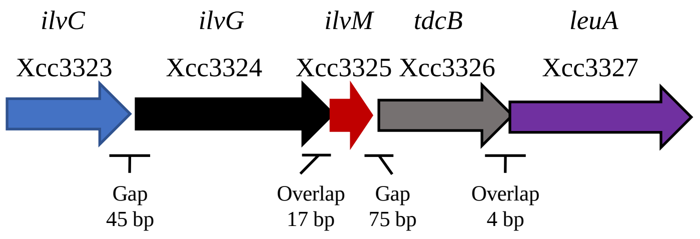

Figure S7. The *ilvCGM-leuA* gene cluster in the genome of Xcc strain ATCC33913.

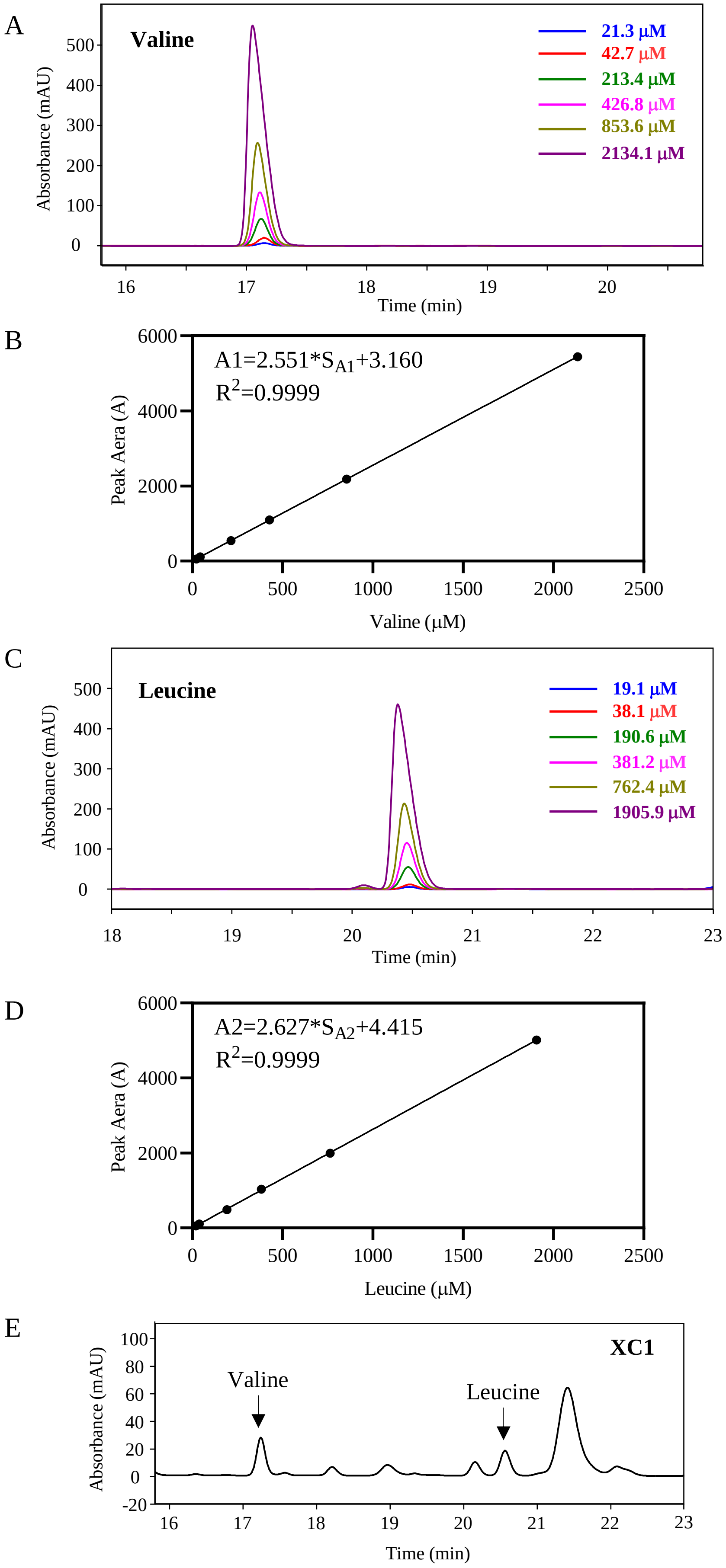

Figure S8. Establishment of a single standard curve for quantification of valine and leucine concentration using the peak aera (A) derived from high performance liquid chromatography (HPLC) analysis. (A) HPLC spectra of valine at 21.3 µM to 2134.1 µM; (B) The plot between the peak area (A1) of the chromatogram and valine concentration (S_{A1}); (C) HPLC spectra of leucine at 19.1 µM to 1905.9 µM; (D) The plot between the peak area (A2) of the chromatogram and leucine concentration (S_{A2}); (E) HPLC analysis of the valine and leucine extract from XC1 XYS cultures at 24 hpi.

Table S1. Bacterial strains and plasmids used in this study

Strain	Properties / characteristics	Reference / source
**Xcc Strains**		
XC1	Xcc wild-type strain, Rif^R	Lab stock
Δ*rpfF*	The *rpfF* in-frame deletion mutant, Rif^R	[1]
Δ*rpfC*	The *rpfC* in-frame deletion mutant, Rif^R	[2]
Δ*rpfB*	The *rpfB* in-frame deletion mutant, Rif^R	[3]
ΔXcc1569	The Xcc1569 in-frame deletion mutant, Rif^R	This study
ΔXcc3275	The Xcc3275 in-frame deletion mutant, Rif^R	This study
ΔXcc1838	The Xcc1838 in-frame deletion mutant, Rif^R	This study
ΔXcc3324	The Xcc3324 in-frame deletion mutant, Rif^R	This study
ΔXcc0206	The Xcc0206 in-frame deletion mutant, Rif^R	This study
ΔXcc0101	The Xcc0101 in-frame deletion mutant, Rif^R	This study
ΔXcc3403	The Xcc3403 in-frame deletion mutant, Rif^R	This study
ΔXcc0354	The Xcc0354 in-frame deletion mutant, Rif^R	Lab stock
ΔXcc2336	The Xcc2336 in-frame deletion mutant, Rif^R	This study
ΔXcc1260	The Xcc1260 in-frame deletion mutant, Rif^R	This study
ΔXcc1791	The Xcc1791 in-frame deletion mutant, Rif^R	This study
ΔXcc0924	The Xcc0924 in-frame deletion mutant, Rif^R	This study
ΔXcc0209	The Xcc0209 in-frame deletion mutant, Rif^R	This study
ΔXcc2217	The Xcc2217 in-frame deletion mutant, Rif^R	This study
ΔXcc2688	The Xcc2688 in-frame deletion mutant, Rif^R	This study
XC1(pBBR)	The strain XC1 harboring the expression plasmid pBBR, Rif^R Kan^R	This study
XC1 (Xcc1569)	The strain XC1 harboring the expression plasmid pBBR-Xcc1569, Rif^R Kan^R	This study
XC1 (Xcc3275)	The strain XC1 harboring the expression plasmid pBBR-Xcc3275, Rif^R Kan^R	This study
XC1 (Xcc1838)	The strain XC1 harboring the expression plasmid pBBR-Xcc1838, Rif^R Kan^R	This study
XC1 (Xcc3324)	The strain XC1 harboring the expression plasmid pBBR-Xcc3324, Rif^R Kan^R	This study
XC1 (Xcc0206)	The strain XC1 harboring the expression plasmid pBBR-Xcc0206, Rif^R Kan^R	This study
XC1 (Xcc0101)	The strain XC1 harboring the expression plasmid pBBR-Xcc0101, Rif^R Kan^R	This study
XC1 (Xcc3403)	The strain XC1 harboring the expression plasmid pBBR-Xcc3403, Rif^R Kan^R	This study
XC1 (Xcc0354)	The strain XC1 harboring the expression plasmid pBBR-Xcc0354, Rif^R Kan^R	This study
XC1 (Xcc2336)	The strain XC1 harboring the expression plasmid pBBR-Xcc2336, Rif^R Kan^R	This study
XC1 (Xcc1260)	The strain XC1 harboring the expression plasmid pBBR-Xcc1260, Rif^R Kan^R	This study
XC1 (Xcc1791)	The strain XC1 harboring the expression plasmid pBBR-Xcc1791, Rif^R Kan^R	This study

Strain	Properties / characteristics	Reference / source
**Xcc Strains**		
XC1 (Xcc0924)	The strain XC1 harboring the expression plasmid pBBR-Xcc0924, Rif^R Kan^R	This study
XC1 (Xcc0292)	The strain XC1 harboring the expression plasmid pBBR-Xcc0292, Rif^R Kan^R	This study
XC1 (Xcc2217)	The strain XC1 harboring the expression plasmid pBBR-Xcc2217, Rif^R Kan^R	This study
XC1 (Xcc2688)	The strain XC1 harboring the expression plasmid pBBR-Xcc2688, Rif^R Kan^R	This study
Δ*ilvC*	The *Xcc3323* in-frame deletion mutant, Rif^R	This study
Δ*ilvC*::*ilvC*	The Δ*ilvC* harboring a single copy of *ilvC* inserted at the attTn*7* site on its chromosome, Rif^R	This study
***E. coli* strains**		
DH5α	*E. coli* F– Φ80lacZΔM15 Δ(lacZYA-argF) U169 recA1 endA1 hsdR17 (rK–, mK+) phoA supE44 λ– thi-1 gyrA96 relA1	Lab stock
S17-1	res$^-$ pro mod$^+$ integrated copy of RP4, mob$^+$	Lab stock
RK2013	Triparental mating helper strain, Kan^R	Lab stock

Plasmids	Properties / characteristics	Reference / source
pK18mobsacB	A mobilizable vector, allows for selection of double crossover in Xcc, Kan^R	[4]
pK18-Xcc1569	Xcc Xcc1569 deletion cassette in pK18mobscaB, Kan^R	This study
pK18-Xcc3275	Xcc Xcc3275 deletion cassette in pK18mobscaB, Kan^R	This study
pK18-Xcc1838	Xcc Xcc1838 deletion cassette in pK18mobscaB, Kan^R	This study
pK18-Xcc3324	Xcc Xcc3324 deletion cassette in pK18mobscaB, Kan^R	This study
pK18-Xcc0206	Xcc Xcc0206 deletion cassette in pK18mobscaB, Kan^R	This study
pK18-Xcc0101	Xcc Xcc0101 deletion cassette in pK18mobscaB, Kan^R	This study
pK18-Xcc3403	Xcc Xcc3403 deletion cassette in pK18mobscaB, Kan^R	This study
pK18-Xcc2336	Xcc Xcc2336 deletion cassette in pK18mobscaB, Kan^R	This study
pK18-Xcc1260	Xcc Xcc1260 deletion cassette in pK18mobscaB, Kan^R	This study
pK18-Xcc1791	Xcc Xcc1791 deletion cassette in pK18mobscaB, Kan^R	This study
pK18-Xcc0924	Xcc Xcc0924 deletion cassette in pK18mobscaB, Kan^R	This study
pK18-Xcc0292	Xcc Xcc0292 deletion cassette in pK18mobscaB, Kan^R	This study
pK18-Xcc2688	Xcc Xcc2688 deletion cassette in pK18mobscaB, Kan^R	This study
pK18-Xcc2217	Xcc Xcc2217 deletion cassette in pK18mobscaB, Kan^R	This study
pBBR1MCS-2	Plasmid for gene complementation, Kan^R	[5]
pBBR1MCS-2-Xcc1569	Xcc1569 cloned in pBBR1MCS-2, Kan^R	This study
pBBR1MCS-2-Xcc3275	Xcc3275 cloned in pBBR1MCS-2, Kan^R	This study
pBBR1MCS-2-Xcc1838	Xcc1838 cloned in pBBR1MCS-2, Kan^R	This study
pBBR1MCS-2-Xcc3324	Xcc3324 cloned in pBBR1MCS-2, Kan^R	This study
pBBR1MCS-2-Xcc0206	Xcc0206 cloned in pBBR1MCS-2, Kan^R	This study
pBBR1MCS-2-Xcc0101	Xcc0101 cloned in pBBR1MCS-2, Kan^R	This study
pBBR1MCS-2-Xcc3403	Xcc3403 cloned in pBBR1MCS-2, Kan^R	This study
pBBR1MCS-2-Xcc0354	Xcc0354 cloned in pBBR1MCS-2, Kan^R	This study

Plasmids	Properties / characteristics	Reference / source
pBBR1MCS-2-Xcc2336	Xcc2336 cloned in pBBR1MCS-2, Kan^R	This study
pBBR1MCS-2-Xcc1260	Xcc1260 cloned in pBBR1MCS-2, Kan^R	This study
pBBR1MCS-2-Xcc1791	Xcc1791 cloned in pBBR1MCS-2, Kan^R	This study
pBBR1MCS-2-Xcc0924	Xcc0924 cloned in pBBR1MCS-2, Kan^R	This study
pBBR1MCS-2-Xcc0292	Xcc0292 cloned in pBBR1MCS-2, Kan^R	This study
pBBR1MCS-2-Xcc2217	Xcc2217 cloned in pBBR1MCS-2, Kan^R	This study
pBBR1MCS-2-Xcc2688	Xcc2688 cloned in pBBR1MCS-2, Kan^R	This study
mini-Tn7T-Gm	a versatile mini-Tn*7* delivery vector mini-Tn7T-Gm, Gm^R	[6]
mini-Tn7-*ilvC*	promoter region and gene cluster of *ilvC* cloned in mini-Tn7T-Gm, Gm^R	This study

References:

1 He YW, Xu M, Lin K, Ng YJ, Wen CM, Wang LH, et al. Genome scale analysis of diffusible signal factor regulon in *Xanthomonas campestris* pv. *campestris*: identification of novel cell-cell communication-dependent genes and functions. *Mol Microbiol*. 2006; **59**: 610-22.

2 He YW, Wang C, Zhou L, Song H, Dow JM, Zhang LH. Dual signaling functions of the hybrid sensor kinase RpfC of *Xanthomonas campestris* involve either phosphorelay or receiver domain-protein interaction. *J Biol Chem*. 2006; **281**: 33414-21.

3 Zhou L, Wang XY, Sun S, Yang LC, Jiang BL, He YW. Identification and characterization of naturally occurring DSF-family quorum sensing signal turnover system in the phytopathogen *Xanthomonas*. *Environ Microbiol*. 2015; **17**: 4646-4658.

4 Schäfer A, Tauch A, Jäger W, Kalinowski J, Thierbach G, Pühler A. Small mobilizable multi-purpose cloning vectors derived from the *Escherichia coli* plasmids pK18 and pK19: selection of defined deletions in the chromosome of *Corynebacterium glutamicum*. *Gene*. 1994; **145**: 69-73.

5 Kovach ME, Elzer PH, Hill DS, Robertson GT, Farris MA, Roop RM 2nd, et al. Four new derivatives of the broad-host-range cloning vector pBBR1MCS, carrying different antibiotic-resistance cassettes. *Gene*. 1995; **166**: 175-176.

6 Choi KH, Schweizer HP. mini-Tn*7* insertion in bacteria with single attTn*7* sites: example *Pseudomonas aeruginosa*. *Nat Protoc*. 2006; **1**: 153-61.

Table S2. Oligonucleotide primers used in this study

Application	Primer and application	Sequence (5' to 3')
Amplification of Xcc Xcc1569 deletion cassette	Xcc1569_del_F1	ctatgacatgattacgaattcCTGATGCGGCGCACGAACTCG
	Xcc1569_del_R1	*CTGCATCGAGTCGGC*ATCCAGTGCAGCCGGCGAC
	Xcc1569_del_F2	GCCGACTCGATGCAGAAGCAGG
	Xcc1569_del_R2	caggtcgactctagaggatccCTGCGTGGTGAAGACGTGGTGG
Amplification of Xcc Xcc3275 deletion cassette	Xcc3275_del_F1	ctatgacatgattacgaattcGTGCTGTGGTGTGGAAGTGTTGCC
	Xcc3275_del_R1	*GCTTGCGGTGGCGTC*GGTAGAACCGGCATAGGCCATCAGG
	Xcc3275_del_F2	GACGCCACCGCAAGCGTACAG
	Xcc3275_del_R2	caggtcgactctagaggatccCCAACCACACTCTGAGTTCAGCCG
Amplification of Xcc Xcc1838 deletion cassette	Xcc1838_del_F1	ctatgacatgattacgaattcCAGGATGGTGAGGCCTTGTGCG
	Xcc1838_del_R1	*CACCCACAGGAACAT*GCGCAACAGCTGATCGCTGG
	Xcc1838_del_F2	ATGTTCCTGTGGGTGACGCTGC
	Xcc1838_del_R2	caggtcgactctagaggatccCTGCAACACGCGGTAATACCAGC
Amplification of Xcc Xcc3324 deletion cassette	Xcc3324_del_F1	ctatgacatgattacgaattcGTCCGCACAACGCATGAACACCTC
	Xcc3324_del_R1	*TGCGTGGGTGGTTGA*CAATGCCGCCACCGCCGTAGA
	Xcc3324_del_F2	TCAACCACCCACGCAACCATCTCA
	Xcc3324_del_R2	caggtcgactctagaggatccCAACTGCAGGCGCAGCGTTTCC
Amplification of Xcc Xcc0206 deletion cassette	Xcc0206_del_F1	ctatgacatgattacgaattcCACATGGAAGTTCGGAGCGAAGGC
	Xcc0206_del_R1	*CGAGGAGGTCTCCAC*GTGGCGCACATGCATCCATTCG
	Xcc0206_del_F2	GTGGAGACCTCCTCGCAGCTGGAG
	Xcc0206_del_R2	caggtcgactctagaggatccGAGCACCGTGTGGCAGCTCG
Amplification of Xcc Xcc0101 deletion cassette	Xcc0101_del_F1	ctatgacatgattacgaattcCCGATGAATGCACTTCCTTCCGC
	Xcc0101_del_R1	*TCCTGGATCAGCGCG*ATCACCACGCAGTTGCCGGC
	Xcc0101_del_F2	CGCGCTGATCCAGGAATCGATCT
	Xcc0101_del_R2	caggtcgactctagaggatccGCGGGATAGGCGTGGTAGCAGTT
Amplification of Xcc Xcc3403 deletion cassette	Xcc3403_del_F1	ctatgacatgattacgaattcGCAGTGGTGCTGGCATCGCTG
	Xcc3403_del_R1	*GGTGAGCAGGCTCAG*GTCGGCGTTGTGGACGTTGGC
	Xcc3403_del_F2	CTGAGCCTGCTCACCTACGACGAC
	Xcc3403_del_R2	caggtcgactctagaggatccGCCGCGGATGTAGCACATGCC
Amplification of Xcc Xcc2336 deletion cassette	Xcc2336_del_F1	ctatgacatgattacgaattcCAGCGATTTGCAGCGGTTGTGACG
	Xcc2336_del_R1	*GTCGCGTCCTGCTGC*CTGGACTGCGCCTCGGTTGGAATG
	Xcc2336_del_F2	GCAGCAGGACGCGACCACACTT
	Xcc2336_del_R2	caggtcgactctagaggatccCTTGCAAATCCCGAATCCCGACTC
Amplification of Xcc Xcc1260 deletion cassette	Xcc1260_del_F1	ctatgacatgattacgaattcGCACGTAACGCCTCATCCAGCG
	Xcc1260_del_R1	*CGGCACGTTGATGCC*CACTTCCAGGCCGCGGAACACATC
	Xcc1260_del_F2	GGCATCAACGTGCCGATCCCG
	Xcc1260_del_R2	caggtcgactctagaggatccGCCTTGCTGCCGTTGAGCACG
Amplification of Xcc Xcc1791 deletion cassette	Xcc1791_del_F1	ctatgacatgattacgaattcGGCCGTCCCGACTTTCTTGG
	Xcc1791_del_R1	*GCCTTGCGCCAGCGG*GCGGCGGGCGACCTTCTC
	Xcc1791_del_F2	CCGCTGGCGCAAGGCATCAAGT
	Xcc1791_del_R2	caggtcgactctagaggatccGCCAGGCCGCCGAAGAACAGGAC
Amplification of Xcc Xcc0924 deletion cassette	Xcc0924_del_F1	ctatgacatgattacgaattcGGAAATCCGCCTGCGCAAGAAGTT
	Xcc0924_del_R1	*GAGTCAGCCCGCCGC*CGGCCAGGGAAAGCAGGGACAG
	Xcc0924_del_F2	GCGGCGGGCTGACTCGTAATCC
	Xcc0924_del_R2	caggtcgactctagaggatccCATCGGTGGCCAGGCGGGTTTC

Application	Primer and application	Sequence (5’ to 3’)
Amplification of Xcc Xcc0292 deletion cassette	Xcc0292_del_F1	ctatgacatgattacgaattcCCACGCCGCGAATTCAACATCCG
	Xcc0292_del_R1	*GAACAGGCGGTCTTC*CGAAGTGCTTGTGTCCGGTGATGC
	Xcc0292_del_F2	GAAGACCGCCTGTTCGCTCTGGC
	Xcc0292_del_R2	caggtcgactctagaggatccGAAACTGTCCAGGCCATCGTGCG
Amplification of Xcc Xcc2217deletion cassette	Xcc2217_del_F1	ctatgacatgattacgaattcGAGCAGCTGCTGACCACACGGCT
	Xcc2217_del_R1	*GCAGGTCGTAGCAGA*TGGTGAAGGTTTCCGGCAGGATCAC
	Xcc2217_del_F2	TCTGCTACGACCTGCGCTTTCCGG
	Xcc2217_del_R2	caggtcgactctagaggatccGCAGGTGTTCCACGACGAGCCG
Amplification of Xcc Xcc2688 deletion cassette	Xcc2688_del_F1	ctatgacatgattacgaattcGTTCCGCGCGGCAGGCATC
	Xcc2688_del_R1	*TGTCGGTGCAGCTGG*GCAAACCGGCCGAAATCTGCGTAC
	Xcc2688_del_F2	CCAGCTGCACCGACACTGCCG
	Xcc2688_del_R2	caggtcgactctagaggatccCTTGGACATGGGTGACCTGGTCGG
Xcc1569 protein expression by pBBR	Xcc1569-F	gataagcttgatatcgaattcGTGTCGTTGCCCGTCTCGCG
	Xcc1569-R	cgctctagaactagtggatccCTGGGCGGCACGAGGCTTC
Xcc3275 protein expression by pBBR	Xcc3275-F	gataagcttgatatcgaattcATGCATACCGCTCTGTCGCGC
	Xcc3275-R	cgctctagaactagtggatccCGTCAGAGTACCGCGAGCAGG
Xcc1838 protein expression by pBBR	Xcc1838-F	gataagcttgatatcgaattcGCTGTCGATCCGCGTCAACTGC
	Xcc1838-R	cgctctagaactagtggatccGACGCCGCTGCTCGATGTAGC
Xcc3324 protein expression by pBBR	Xcc3324-F	gataagcttgatatcgaattcATGAACACCTCCGCACACAGCA
	Xcc3324-R	cgctctagaactagtggatccGCCGTTCGGTCATGCCGATCAC
Xcc0206 protein expression by pBBR	Xcc0206-F	gataagcttgatatcgaattcGTGGCGAAGAAGAAGACGGTGGC
	Xcc0206-R	cgctctagaactagtggatccGTGCGATCCGAAAGGAGACGTGC
Xcc0101 protein expression by pBBR	Xcc0101-F	gataagcttgatatcgaattcATGAATGCACTTCCTTCCGCCAAGT
	Xcc0101-R	cgctctagaactagtggatccGGACCTACCACATGCCGCTCG
Xcc3403 protein expression by pBBR	Xcc3403-F	gataagcttgatatcgaattcATGCCACGTTTCCCCGACCAG
	Xcc3403-R	cgctctagaactagtggatccCGCTCGTTTGAACGGCAGTACG
Xcc0354 protein expression by pBBR	Xcc0354-F	gataagcttgatatcgaattcATGCATGCGCTCCCCAGCAT
	Xcc0354-R	cgctctagaactagtggatccCAGTTCCGTGCGGCAGGGTAAG
Xcc2336 protein expression by pBBR	Xcc2336-F	gataagcttgatatcgaattcATGTCCTACGACACCGTCAATCCGG
	Xcc2336-R	cgctctagaactagtggatccCGCTGCTCTTGCAAATCCCGAATC
Xcc1260 protein expression by pBBR	Xcc1260-F	gataagcttgatatcgaattcATGAGCGAATCCGTCCCACGC
	Xcc1260-R	cgctctagaactagtggatccCAGTTGGATCGCTGCGTTCATGG
Xcc1791 protein expression by pBBR	Xcc1791-F	gataagcttgatatcgaattcATGTCCGCTGACCTGCTCAAGG
	Xcc1791-R	cgctctagaactagtggatccGTGGTACCAATAGGCGCAACGAGC
Xcc0924 protein expression by pBBR	Xcc0924-F	gataagcttgatatcgaattcATGCGCACACCCATCCTGTCC
	Xcc0924-R	cgctctagaactagtggatccGATGCCTGCATCGCCTCCACG
Xcc0292 protein expression by pBBR	Xcc0292-F	gataagcttgatatcgaattcATGCAACTCCCCGCGCAGTG
	Xcc0292-R	cgctctagaactagtggatccGGTGAGGCGCGACCACCATG
Xcc2217 protein expression by pBBR	Xcc2217-F	gataagcttgatatcgaattcATGCACGACCTGCGCATTTCC
	Xcc2217-R	cgctctagaactagtggatccCAGCTGCTCCAGGCGGAAGC

Application	Primer and application	Sequence (5’ to 3’)
Xcc2688 protein expression by pBBR	Xcc2688-F	gataagcttgatatcgaattcATGAAGATTGCCGTTGCCAAATACC
	Xcc2688-R	cgctctagaactagtggatccGGAAGCGCTACGCATCCAC
RT-PCR analysis	*ilvC-ilvG*-F	GCAGGCCGATCTGGAGCACCC
	ilvC-ilvG-R	CCATCGGCACCGAGTCGAGCATG
	ilvM-tdcB-F	CTTCCGCCCGTGCGCCATCC
	ilvM-tdcB-R	CGCCCAACCGATACGCCGACC
ilvC complementation	*ilvC* rec_F	catgagctcactagtggatccCGCAATTGGTGACGCCGGTTG
	ilvC rec_R	cgaggtaccgggcccaagcttGAGGTGTTCATGCGTTGTGCGGAC
